# The effectiveness of dialectical behaviour therapy training: a quantitative systematic review using Kirkpatrick’s four-level model

**DOI:** 10.1186/s40479-026-00344-4

**Published:** 2026-04-24

**Authors:** Melanie Harris, Carolien Lamers, Michaela Swales

**Affiliations:** North Wales Clinical Psychology Programme, School of Human and Behavioural Sciences, Bangor University, Bangor, UK

**Keywords:** Dialectical behaviour therapy, DBT, Training, Implementation, Review

## Abstract

Effective therapist training is critical to the successful implementation of Dialectical Behaviour Therapy (DBT) in routine clinical practice. Although various training approaches exist, their outcomes have not been comprehensively synthesised. This systematic review examined the effectiveness of DBT training across different delivery formats and identified key factors associated with training success. Four databases (Medline, CINAHL, PsycInfo, and PubMed) were searched for quantitative studies, and the quantitative components of mixed-methods studies, evaluating training outcomes according to Kirkpatrick’s Four-Level Model. Twenty-four studies met inclusion criteria. Six priority outcomes were assessed for certainty using the GRADE approach. Overall, DBT training was well received and associated with positive effects on therapists’ knowledge, self-efficacy, attitudes, and self-reported DBT use. More intensive training models with implementation support were linked to improved knowledge acquisition, motivation, self-reported DBT use, and, in some cases, client outcomes. The studies were highly heterogeneous in design and measures, few reported effect sizes, and most carried moderate risk of bias. As a result, the overall certainty of evidence was generally low. These findings highlight the potential effectiveness of DBT training while emphasising the need for more rigorous, standardised evaluations to strengthen the evidence base.

**Supplementary information:**

The online version contains supplementary material available at 10.1186/s40479-026-00344-4.

## Introduction

Dialectical Behaviour Therapy (DBT) is a cognitive-behavioural treatment, developed by Marsha Linehan in the 1980s, for women with a diagnosis of borderline personality disorder (BPD) [1]. DBT is recommended by the National Institute for Health and Care Excellence [2] for the treatment of BPD, and its effectiveness has been demonstrated across several reviews [3–8]. There is also growing evidence supporting DBT for other clinical populations and settings, including adolescents, individuals with eating disorders, and forensic populations [9–11].

DBT is underpinned by a dialectical philosophy that balances acceptance and change strategies [1]. Comprehensive DBT is a multi-component treatment that includes individual therapy, group skills training, telephone coaching, and a therapist consultation team [1]. Services, however, sometimes deliver only some components, such as skills training alone [12]. Additionally, several evidence-based adaptations of DBT have been developed for specific populations, such as DBT for Complex Post Traumatic Stress Disorder (DBT-PTSD) [13] and DBT-Prolonged Exposure (DBT-PE) [14].

Research comparing data from effectiveness studies and randomised controlled trials (RCTs) suggests that, while DBT maintains positive outcomes in routine clinical practice, effect sizes are often smaller and more variable [15]. One suggested explanation for this discrepancy is the level of therapist training [15]. Following the publication of the DBT manual and the first RCT, Linehan developed a structured training approach to support services in implementing a comprehensive DBT programme [16]. The DBT Intensive Training™ Model (DBT-ITM) is a team-based training format and is widely regarded as the gold standard for DBT training [17]. There is evidence that DBT-ITM leads to the successful initiation of DBT programmes [18]. Nevertheless, DBT-ITM is resource-intensive and costly, and to enhance the broader dissemination of DBT, alternative training formats have been explored [19].

Research by Harned et al. [20] suggests that greater adherence to DBT is associated with greater reductions in client suicidal behaviours, emphasising the important role of therapist training in enhancing clinical outcomes; thus, evaluating the effectiveness of DBT training programmes is critical to ensure that training efforts translate into meaningful clinical practice. Evaluations typically assess trainee satisfaction, as well as the extent to which training improves knowledge, skills, confidence, and the ability to deliver DBT competently. Given the diversity of available DBT training formats, including workshops, modular courses, and online training, it is important to systematically assess which approaches are effective, and in what contexts. Systematically reviewing DBT training outcomes can help identify which methods are associated with improvements in therapist competencies, DBT use, and ultimately client outcomes. To date, no comprehensive systematic review has synthesised the available evidence of DBT training outcomes across different training formats. This review addresses this gap and can be used to inform services and future DBT training initiatives.

To systematically organise and synthesise outcomes reported across DBT training studies, Kirkpatrick’s Four-Level Training Evaluation Model [21] was selected as the guiding framework for this review. The model provides a comprehensive structure for evaluating training effectiveness across four outcome levels: reaction, learning, behaviour change, and results (e.g., client or organisational outcomes). It is widely used across diverse fields and is particularly useful for synthesising heterogeneous evidence where outcomes are reported at different stages of training impact [22]. Although critiques of the model suggest that its linearity oversimplifies the complex processes linking training to organisational outcomes, it remains a pragmatic and widely accepted structure for training evaluations [22, 23]. Recognising these limitations, this review treats the four Kirkpatrick outcome levels as separate domains and will only highlight links between them where there is empirical evidence, rather than assuming a linear or causal sequence.

This review aimed to systematically synthesise the outcomes of DBT training programmes for mental health professionals in routine clinical practice, to answer the research questions:

1. What is the effectiveness of Dialectical Behaviour Therapy (DBT) training, as classified by Kirkpatrick’s Four-Level Training Evaluation Model [21]?
  - Level 1 (Reaction): How do participants perceive and react to DBT training programmes? (e.g., satisfaction, engagement)
  - Level 2 (Learning): What is the effectiveness of DBT training on participants’ knowledge, confidence, motivation, intentions or attitudes?
  - Level 3 (Behaviour): What is the effectiveness of DBT training on behaviour change in participants’ clinical practice?
  - Level 4 (Results): What is the effectiveness of DBT training on organisational or system-level outcomes? (e.g., client outcomes, implementation outcomes)
2. What training components (e.g., duration, format) are associated with better outcomes at each level?

A preliminary search of PROSPERO and databases (Medline (EBSCO), CINAHL (EBSCO), Psycinfo (Proquest), PubMed (NCBI)) was undertaken and no existing or ongoing systematic reviews on the topic were identified.

## Method

The review protocol was registered with PROSPERO (Registration Number CRD420251037948). The systematic review followed the Preferred Reporting Items for Systematic Reviews and Meta-Analyses guidelines [24] and was conducted in accordance with the Joanna Briggs Institute (JBI) methodology for effectiveness systematic reviews [25].

### Search strategy

Four electronic databases were searched in February 2025: Medline (EBSCO), CINAHL (EBSCO), PsycInfo (ProQuest), and PubMed (NCBI). The search terms were: (implement* or train* or workshop* or teach* or learn* or student* or develop* or educat* or course* or disseminat*) and (dialectical behaviour or dialectical behavior or dialectical behavioural OR dialectical behavioral). Searches were limited to English language and peer reviewed. No date restrictions were applied. The full search strategy is presented in Supplementary File 1.

### Study selection

The focus of this review was on studies evaluating DBT training with mental health professionals. The following criteria were applied to the retrieved papers:

### Population

Eligible studies involved practicing mental health professionals (e.g., therapists, clinicians), working in routine clinical services, who had participated in DBT training. Student and trainee-only samples were excluded as training approaches and implementation challenges differ from those in routine clinical services [26]. Studies reporting on client outcomes that were attributed to DBT training were also eligible.

### Intervention

Eligible studies were papers which evaluated or reported outcomes following standard DBT training (e.g., workshops, courses, structured training packages). To be eligible, the DBT training had to be educational in nature, aiming to teach professionals to deliver or implement DBT. Studies were excluded if the training was delivered as an intervention or focused solely on supervision or practice development.

Training was considered to be standard DBT if it was based on the original Linehan [1] model. This included trainings designed to implement either comprehensive DBT or skills-only DBT, provided that they covered the four core modules of mindfulness, emotion regulation, distress tolerance, and interpersonal effectiveness. Trainings focused on teaching specific DBT skills were also included if they were grounded in the Linehan [1] model. Adapted versions of DBT, such as DBT-PE or DBT-PTSD, were considered standard DBT if they were regarded as extensions of the original model. In contrast, trainings that integrated DBT with another treatment approach or were based on fundamentally different models, such as Radically Open DBT, were excluded. When it was unclear whether a training met the criteria for standard DBT, the third author, who is an expert in DBT training, was consulted.

### Comparison

Different comparators were required for each level of Kirkpatrick’s Four-Level Training Evaluation Model [21] to ensure that outcomes were attributable to the training and appropriate for the outcome level. Studies were eligible if they included a comparator consistent with one or more levels of the Kirkpatrick model, and where DBT training functioned as an active variable under investigation. Studies were excluded if the comparator focused solely on the effects of DBT as a treatment. Acceptable comparators included pre–post training assessments, control groups, comparisons to benchmarks, variations in training format or intensity, and service-level comparisons. Specific inclusion criteria by level (Reaction, Learning, Behaviour, and Results) are detailed in Supplementary File 2.

### Outcome

Eligible studies reported one or more outcomes aligned with Kirkpatrick’s model of training evaluation, which classifies outcomes into four levels:

1. Reaction: participant satisfaction and engagement with the training.
2. Learning: changes in participant knowledge, attitudes, motivation, intentions or confidence.
3. Behaviour: changes in participant behaviour (e.g., DBT use).
4. Results: client or organisational outcomes (e.g., symptom improvement, implementation).

### Types of studies

Quantitative and mixed-methods studies were eligible for the review, provided that the research methods were clearly reported and outcomes could be reliably extracted. Level 2 to 4 outcome comparators required inferential statistics to allow for an effectiveness interpretation. Level 1 and cost outcomes only required descriptive statistics due to the nature of the outcome. Conference abstracts, dissertations, non-peer-reviewed articles, and grey literature were excluded. Papers included in the review were limited to full-text, peer-reviewed articles published in English.

### Selection process

Retrieved records were exported into the reference management software Cadima. The first and third authors independently applied the inclusion and exclusion criteria to 10% of the identified records at both the title/abstract and full-text stages to assess consistency in applying the criteria. At the abstract screening stage, the Kappa value was 0.72, with disagreement on only three records, all of which were resolved through discussion. The first author then screened the remaining titles and abstracts, followed by full-text records. For records with ambiguous or borderline eligibility, the reviewer consulted with the third author to reach consensus, ensuring consistent application of the criteria throughout the review process.

To identify additional relevant studies, backward and forward citation searching of included studies was conducted by the first author in May 2025. Google Scholar was used for forward citation searching. Titles of citing and cited records were initially screened for relevance. Where titles had not already been screened and appeared potentially relevant, abstracts and full texts were reviewed to determine eligibility.

### Data collection

The first author independently extracted data, using Microsoft Excel, informed by the data extraction template (Supplementary File 3). The second author evaluated the extraction of four of the studies to ensure accuracy and consistency.

### Quality assessment

The included papers were quality-assessed using the appropriate tool for each study design. Quality assessment tools included JBI checklists for randomised controlled trials [27], cohort studies [28], quasi-experimental studies [29], analytical cross-sectional studies [28]. One study [30] required a CASP checklist for descriptive/cross-sectional studies [31] as it only presented Level 1 descriptive data, whereby there was no appropriate JBI tool.

The first and second authors independently assessed the quality of four of the studies. While no formal inter-rater reliability statistics were calculated, discrepancies were resolved through discussion to ensure consistency in evaluation. The first author then independently completed the quality appraisal for all remaining papers.

### Certainty of evidence

In line with the GRADE (Grading of Recommendations, Assessment, Development and Evaluation) [32] and JBI approach to assessing certainty of evidence [25], six key outcomes were prioritised for formal grading based on their relevance to the research questions and importance to decision-makers. Certainty of evidence refers to the degree of confidence in the effect estimates for each outcome. The outcomes, graded by the first author, were: knowledge, confidence/self-efficacy, adherence, use of DBT in clinical practice, implementation, and suicide and self-harm client outcomes. Other reported outcomes were synthesised narratively but were not formally graded. To account for variation in study designs, and to answer both research questions, GRADE assessments were conducted separately for different comparisons (e.g., pre–post, intensive vs non-intensive, and training vs control). Studies could contribute to more than one comparison depending on the outcomes and design.

### Data synthesis

The review utilised a framework synthesis, guided by a pragmatic epistemological stance [33]. It was anticipated that the review would retrieve papers with a heterogenous selection of methodologies and outcomes. While individual studies may provide partial insights into the outcomes of DBT training, using a framework synthesis, their findings could be systematically organised within an established conceptual framework, Kirkpatrick’s Four-Level Training Evaluation Model [21], to build cumulative understanding.

## Results

### Study selection

**Fig. 1:**
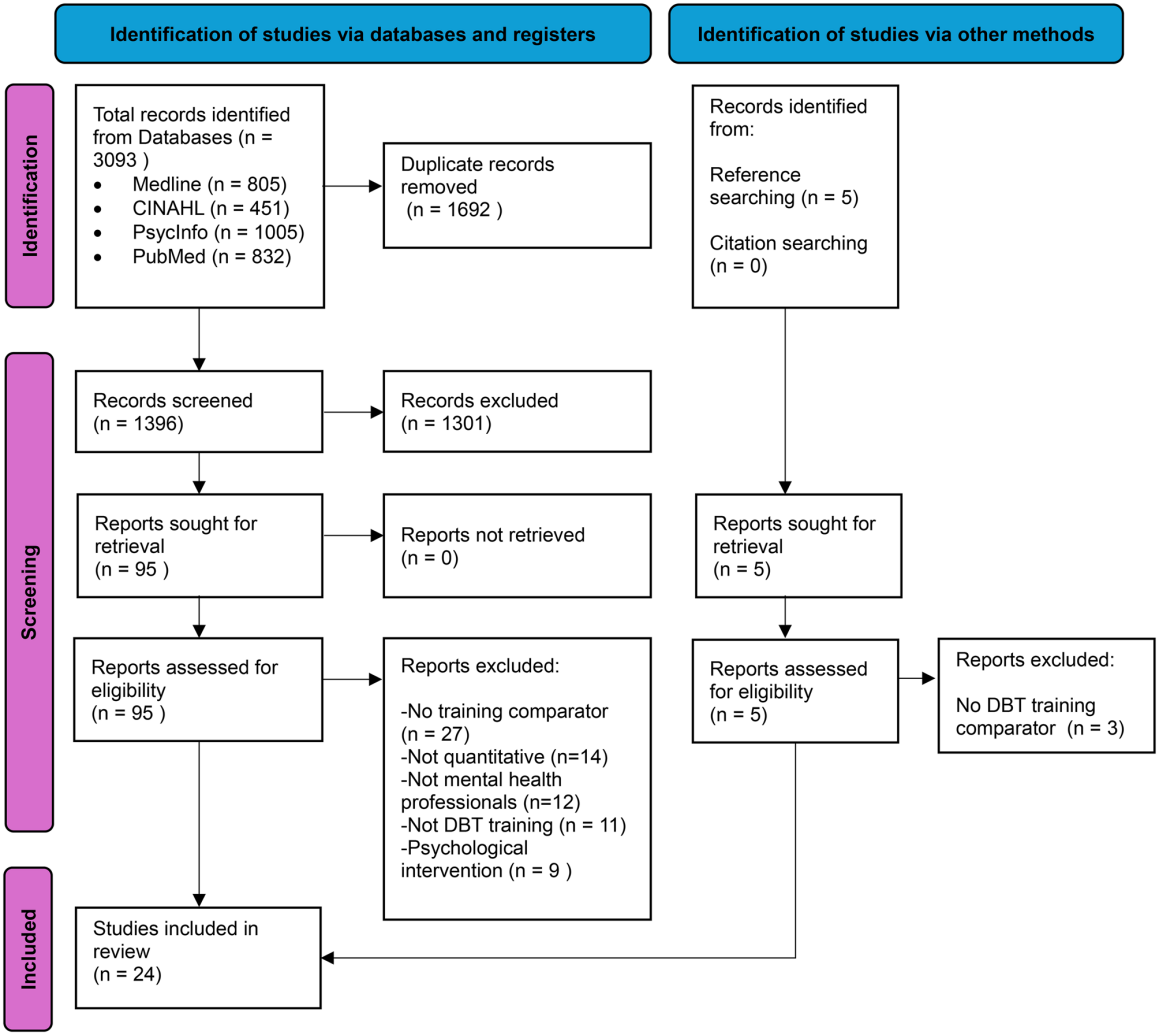
PRISMA flowchart of study selection process

As shown in Fig. 1, the database searches retrieved 3,093 records. After removing 1,697 duplicates, 1,396 records remained for title and abstract screening. Of these, 95 were selected for full-text review and 22 studies met the eligibility criteria and were included in the evidence synthesis. The main reasons for exclusion at the full-text stage were no comparator with an active training variable, the training aim was not to teach DBT delivery, psychological intervention rather than training, no quantitative data, not standard DBT training, not routine clinical services. Supplementary File 4 provides examples of excluded studies and reasons for exclusion.

Citation searching identified five additional studies through backward citation searching, of which two met eligibility criteria and were included in the review. No studies identified through forward citation searching were retrieved for full-text review.

### Study characteristics

Table 1 provides an overview of all included studies. Studies were highly heterogeneous. They varied in terms of design: ten employed observational repeated measures (with follow-up periods ranging from 0 to 18 months); five used quasi-experimental designs; three were RCTs; and two were cross-sectional. Other designs included secondary data analyses, retrospective cohort comparisons, observational studies with benchmark comparisons, and descriptive evaluations.

**Table 1:** Study characteristics and outcomes

Author (Year)	Country, service context, DBT adaptions	Study design/method	Population	Training details (format, duration, provider)	Comparator(s)	Outcomes (Kirkpatrick Levels)
Kauth et al. (2017) [30]	U.S., Veterans Health Administration	Descriptive post-training evaluation study	35 mental health leaders and clinicians at ten VHA medical centres; 74% female; 37% psychologists, 43% social workers, 6% nurses, 3% physicians, 3% addiction workers.	Six web-based DBT skills modules (six months access; Behavioral Tech Research) and given DBT skills manual. Plus ‘facilitation’: monthly conference call with DBT skills champion who helped set deadlines and training goals. The two champions received extra external training (over 9 months; from the paper authors).	No comparator- descriptive 6 weeks post-training satisfaction. *N = 33*	**Satisfaction (Level 1):** −88% satisfied with online modules; 97% reported learning new information; 73% had difficulty completing modules during working hours. −72% did not find having the skills manual helpful. −84.8% satisfied with monthly facilitation calls; 79% viewed facilitation calls as a beneficial use of time; 82% said calls helped discuss module content with DBT therapists; 88% said calls helped discuss DBT group work with peers/therapists. −88% rated facilitators as encouraging; 94% said facilitators were responsive to questions; 91% felt facilitators provided structure but allowed self-paced progress. **Attendance (Level 1):** -Approximately 74% of participants attended every call.
McCay et al. (2017) [34]	Canada, community setting working with street involved youth DBT-A- 12 weeks	Observational repeated measures	18 community agency staff; 77.8% female; 56.25% youth workers, 31.25% social workers, 12.5% other; none of the staff had DBT experience.	Multi-component dynamic training over 4–6 months: -Self-directed online DBT training programme (32 hours; Behavioral Tech) - DBT webinars (8 weekly 1.5 hours = 12 hours; expert led) -Self-study Skills Training Manual -Self study manuals outlining study’s specific protocols -Weekly one-hour peer consultation provided throughout.	Pre vs post-training vs 4–6 months post-training vs 12–16 months post-training.	**Attendance (Post-Training Skills Use Questionnaire; Level 1):** -Therapists attended on average 7.79% (*SD* = 0.41) of webinars; 82.4% of therapists completed the self-directed online training; 17.6% of therapists completed 75% of the online training. -Barriers to completing training included technical problems and lack of time/demands at work. **DBT Skills Knowledge Test (Level 2):** -Knowledge increased pre training to post-training* and was maintained at both follow-ups. **Confidence and motivation to use DBT skills (BAQ; Level 2):** -Increased pre- to post-training*; decreased from post-training to 4–6 months follow-up*; increased from 4 to 6 months to 12–16 months follow-up*.
Ashworth et al. (2016) [35]	U.K., medium-secure psychiatric hospital for learning disabilities. “I can feel good” DBT skills programme	Mixed methods; observational repeated measures	11 ward staff; 90.9% male; 55% healthcare workers, 45% nurses.	“I can feel good” staff awareness training (5 hours; led by a trainee Forensic psychologist and clinical psychologist who were running the programme).	Pre vs post training	**Reaction (scale from 1 [poor] to 5 [excellent]; Level 1):** -Presentation Quality: M$$ \cong $$4.6 -Facilitator knowledge: M$$ \cong $$4.7 -Relevance to your job: M$$ \cong $$4.7 -Usefulness: M$$ \cong $$4.6 -Enjoyable M$$ \cong $$4.1 **Motivation (Level 2):** -Increase in score from pre- to post-training*. **Knowledge (Level 2):** -Increase in score from pre- to post-training*. **Confidence (Level 2):** -Increase in score from pre- to post-training*. **Perceived behaviour change (Level 3):** -No significant change from pre-training to one month follow-up. -Descriptive increases were seen for encouraging group support, encouraging group attendance, supporting with homework, modelling, coaching, language; language showed a particular increase; supporting skill rehearsal showed no change.
Hawkins & Sinha (1998) [36]	U.S., Department of Mental Health and Addictions Services (DMHAS), multiple sites and disciplines	Cross-sectional correlational + longitudinal repeated measures subgroup	109 clinicians (majority social workers, also nurses, psychologists, psychiatrists); mostly experienced; 6.4% trainees	DMHAS DBT training initiative: seminars/workshops, on-site training, consultations, 2 × 5-day intensive workshops; expert DBT consultation. Sites varied in how much training they had received at the time of initial exam (from none to a year), thus training varied from person to person.	DBT naïve vs trained Psychologists vs other clinicians Workshop attendees vs non-attendees	**Knowledge (DBT knowledge exam; Level 2):** -Trained clinicians scored higher than untrained*. -Psychologists scored higher than other clinicians*, education correlated with score (*r* = 0.29), but without psychologists, education was not correlated. -Behaviour therapy background was not correlated with exam score. -Five-day workshop completers scored higher than non-attendees, who also had more previous training. -Reading DBT materials (*r* = 0.59), peer consultation (*r* = 0.63), and study group attendance (*r* = 0.65) strongly predicted knowledge; combined training types led to higher scores. -Practice alone without structured learning did not predict better scores. -Repeat exam scores improved significantly with additional training (*r* = 0.58), especially expert consultation (*r* = 0.57), reading (*r* = 0.61), and study groups (*r* = 0.65).
Holbrook et al. (2022) [37]	U.S., five residential treatment settings	Mixed methods; observational repeated measures	58 staff working with individuals with serious mental illness, chemical dependency, and/or forensic involvement.; 82.8% female; 72.7% African American.	**Four-phase training model:** **Phase 1:** Staff Training; didactic and experiential training; Linehan-trained trainer; 24 hours for administrative and line staff concurrently + 3 hours for group facilitators. **Phase 2:** Service design. **Phase 3:** Implementation; coaching and fidelity monitoring. **Phase 4:** Maintenance; monthly team consultation with DBT trainer. Train-the-trainer model used with a staff member.	Pre vs post ‘24-hour didactic training’ Staff turnover data year prior to DBT training vs year following training	**Self administered DBT Knowledge Assessment Quiz (Level 2):** -Scores increased from pre-to post-training*; education had a significant effect*; age and length of tenure were not significantly correlated with total quiz scores. −77.6% scored 83.2% or higher at post-training. **Staff turnover (Level 4):** -Overall staff turnover rate decreased*; 4/5 programmes decreased, one of which significantly decreased. -Total agency staff turnover rates increased slightly.
Herschell et al. (2014) [38]	U.S., community mental health centres	Longitudinal observational repeated measures	64 therapists; 78% female, 95% “Caucasian”, 36% professional counsellors, 36% social workers, 13% psychologists, 13% registered nurses.	DBT-ITM (Behavioral Tech) over 18 months: −2-day treatment overview (+1-day administration overview) -Two 5-day trainings -One 2-day training -Weekly phone consultation.	Pre vs post training vs follow-ups (up to 8 months post-training)	**Attitudes towards consumers with BPD (Level 2)** -Therapists with lower baseline attitudes improved most (*r* = −0.69)*; job role, education, and experience had no impact. **Confidence in the effectiveness of DBT (Level 2):** -Confidence increased over training*. -Therapists with bachelor’s degrees or less improved more than those with master’s degrees or higher*. -More experienced therapists had higher baseline confidence*. -Those with lower baseline confidence improved most (*r* = −0.46)*. **Use of DBT components (Level 3)** -Increased overall*, especially skills training*, treatment targets*, diary cards*, and dialectical strategies*. -More experienced therapists used more DBT at baseline*. -Lowest baseline users showed greatest gains (*r* = −0.29)*. -Increases in attitudes (*r* = 0.30)* and confidence (*r* = 0.45)* linked to greater DBT use. -No change in reported ancillary treatments, but there was a trend of less client emergency room visits and hospitalisations.
Dimeff et al. (2009) [39]	U.S., drug treatment and mental health treatment providers	RCT	150 clinicians; 69.8% female; 80.7% “Caucasian”; 22.8% psychologists, 22.8% social workers, 10.8% counsellors.	Three training methods teaching four DBT skills modules and skills coaching: -Instructor-led training (ILT; Behavioral Tech)- 2-day workshop -Manual training (TM)- DBT skills training manual + study guide -Online training (OLT; Behavioral Tech)- approximately 20 hours to complete.	ILT vs TM vs OLT	**Satisfaction (Level 1):** -OLT & ILT rated higher than TM on style & presentation (*d* = 1.3 & 1.55)*. **Barriers to use (Level 1):** -Similar across groups; TM had more condition-specific barriers (*d* = 0.83)*. **Training completion (Level 1):** -No significant differences between conditions. **Knowledge (DBT skills knowledge and application test; Level 2):** -All improved over time* -OLT showed greater knowledge gains than ILT (*d* = 0.37, 0.44)* and TM (*d* = 0.52-0.51) * post-training and 90-day follow-up. **Self-efficacy (BAQ; Level 2):** -Improved for all*. -OLT (*d* = 0.54)* and ILT (*d* = 0.68)* outperformed TM post-training, but no difference at 90 days. **Skills application (Level 3):** -No group differences at 90 days, but ILT (*d* = 0.72)* and TM (*d* = 0.71)* groups referred to materials more than OLT. **Role play adherence/competence (Level 3):** -All improved*; no differences.
Dimeff et al. (2011) [40]	U.S., community mental health and drug agencies	RCT	132 clinicians; 74.2% female; 82.6% “Caucasian” 41.7% mental health therapists.	Two training methods on crisis survival strategies and one control method (up to 2.5 hours for each): -Manual training (TM) - DBT skills training manual crisis survival pages + handouts -DBT E-learning course (e-DBT; Behavioral Tech) -e-Control: ‘Care of the Client with Borderline Personality Disorder’ online course.	TM vs e-DBT vs e-Control	**Satisfaction (Level 1)** -e-DBT rated more acceptable than TM* (*d* = 0.65, 0.8). -e-DBT (*d* = 1.21, 1.34)* and TM (*d* = 0.56, 0.61)* rated more acceptable than e-Control. -e-DBT (*d* = 0.64, 0.75)* and e-Control (*d* = 0.66, 0.37)* rated more usable than TM. -No significant differences between e-DBT and e-Control on useability. **Time in training (Level 1):** -e-DBT spent more time in training than TM (*d* = 2.11)* and e-Control (*d* = 1.79)*. -No significant difference between Manual and e-Control. **Knowledge (Distress Tolerance DBT skills knowledge and application test Level 2):** - All improved over time*; TM (*d* = 2.84, 1.51)* and e-DBT (*d* = 3.48, 1.90)* outperformed e-control. -No significant difference between TM and e-DBT post-training; e-DBT outperformed TM at 15-week follow-up (*d* = 0.36)*. **Self-efficacy (Level 2):** -Increased across groups*. -e-DBT outperformed e-Control (*d* = 0.76, 0.83)*. -No significant differences between e-DBT and TM at any time point. -TM decreased over time. **Motivation (Level 2):** - All groups started high and remained high; e-Control decreased* at post-training and follow-up to level of e-DBT and TM. - TM had higher motivation than e-Control at 15 weeks (*d* = 0.52)*. **Skills application (Level 3):** -e-DBT led to consistently higher skills use than TM* and e-Control*. TM showed short-term gains, but effects declined. e-Control had the lowest and least sustained skills use.
Dimeff et al. (2015) [41]	U.S., clinicians providing counselling and/or mental health services to individuals with BPD with/without substance abuse problems	RCT	172 clinicians; 76.2% female; 78.5% “Caucasian”; 21.5% drug workers, 16.3% social workers, 20.9% mental health counsellors; 85.3% no prior DBT experience.	Three training methods on validation and chain analysis strategies: -Instructor-led training (ILT) − 2 days, 12 hours, expert-led with active and passive learning -Manual training (TM) - DBT skills training manual pages + study guide -Online training (OLT; Behavioral Tech)- approximately 12 hours to complete over 30 days.	ILT vs TM vs OLT	**Satisfaction (Level 1)** -ILT rated more satisfactory than OLT and TM*. **Barriers to use (Level 1):** -No significant difference across conditions. **Training completion (Level 1):** -ILT (98%) completed more of their training than TM (89%)*. -No significant differences between OLT (92%) and other conditions. **Knowledge (Level 2):** - All improved over time*; OLT had higher gains than TM (*d* = 0.62, 0.46)* and ILT (*d* = 0 0.49, 0.59)*, especially for validation strategies*. **Self-efficacy (BAQ; Level 2):** -Increased across groups*, no difference across groups. -ILT higher than TM (*d* = 0.96, 0.84)* and OLT (*d* = 0.80, 0.89)* at all time points; no differences between OLT and TM may reflect baseline differences. **Motivation (BAQ; Level 2):** - Decreased over time for all*. -ILT higher than TM (*d* = 0.52, 0.67)* and OLT (*d* = 0.45)* at post-training; ILT > TM for chain analysis motivation (*d* = 0.59, 0.82)*. **Skills application (Level 3):** -Strategy use increased post-training*, no group differences. **Role play adherence/competence (Level 3):** -Improved post-training* with no group differences.
Haynos et al. (2016) [42]	U.S., child and adolescent residential psychiatric treatment facility (inpatient)	Observational repeated measures	22 mental health nurses; some worked on a comprehensive DBT residential unit, others worked on units where DBT skills coaching wasn’t a focus. No staff had previous DBT training.	DBT skills coaching training; 6 × 2-hour interactive training sessions, over 12 weeks, delivered by DBT experts.	Pre vs post	**Knowledge (DBT skill knowledge test; Level 2):** -Increase from pre-training (*M* = 63.27; *SD* = 13.8) to post-training (*M* = 74.21; *SD* = 15.68)*. **Attitudes toward BPD (ABPDQ; Level 2):** -Decrease from pre- to post-training*. **Burnout (CBI; Level 3):** -Decrease in personal* and work burnout* subscales; no significant reductions in the client burnout subscale. -All subscale scores correlated with Stigma Towards BPD at pre- (*r* = 0.70-0.72)* and post- (*r* = 0.62-0.79)* training. -Higher pre-training stigma predicted greater client burnout later*. DBT knowledge did not predict client burnout.
Navarro-Haro et al. (2024) [43]	Spain and Latin America, range of mental health services	Observational repeated measures	274 participated. 242 completed pre- measures (88.32%). 61 (25.21%) completed post- measures. 76.4% female, 77.7% Latin America, 22.3% Spain; 69% psychologists, 42.6% private clinics; 20.2% had no previous DBT training.	DBT-ITM (DBT Iberoamérica); two 5-day online trainings, occurred over 5 Saturdays. 6–9 months implementation period between two parts, with 5 sessions of mentoring.	Pre vs post	**Concerns about treating suicidal clients scale (CATSP; Level 2):** -No pre–post differences overall. -At subscale, training and competence concerns decreased after training*; no other subscale change. -Women had more legal concerns than men* at pre- and post-training. **Self-efficacy in assessing and managing suicide risk scale (SETSP-S; Level 2)** -Significant increase post-training*. -Higher in psychiatrists vs psychologists* at pre- and post-training. **Confidence and motivation in applying DBT (BAQ; Level 2):** -No significant difference change to motivation. -Confidence in applying DBT increased*. -Correlated with self-efficacy (*r* = 0.43)* and DBT implementation (*r* = 0.33)*. **Burnout (CBI; Level 3):** -No significant change. -Burnout scores correlated with perceived structural and administrative barriers (*r* = 0.37)*. **Implementation (Program Elements of Treatment Questionnaire; Level 4):** -Increases in group skills training*, consultation team use*, phone coaching*, and mindfulness*; mindfulness showed the most consistent improvement; no change in individual therapy use. -Higher implementation among men and Latin American clinicians. -More reported team barriers (*r* = −0.33)* and higher client caseload (*r* = −0.33)* were linked to lower implementation.
Tan et al. (2023) [44]	Singapore, adult outpatient public psychiatric hospital	Mixed-methods, quasi-experimental	14 practising psychologists (7 in DBT training, 7 in control); all female, Mean age: 32.86.	DBT-ITM part one (5 days; Behavioral Tech). Control group had no DBT training.	Matched control group (no training)	**Attitudes towards BPD (ABPDQ; Level 2):** -Acceptance toward BPD increased more in DBT group vs. control (η^2^ = 0.30)*. -No time X condition differences for Enjoyment, Security, Purpose, Enthusiasm. **Burnout (CBI; Level 3):** - No significant differences. **Therapeutic alliance (Working Alliance Inventory-Short Revised Therapist; Level 3):** -No significant differences.
Bender et al. (2023) [45]	U.S., services not reported	Longitudinal observational repeated measures	31 mental health clinicians; 87.1% female, 54.8% White, 32.3% Hispanic; highly educated, majority earned a master’s degree (74.2%) or doctorate (22.6%).	Comprehensive DBT training (expert DBT trainer): 5 days (40 hours).	Pre vs post training vs 6-month follow-up *N* = 16	**DBT knowledge (Level 2):** -Large improvement in knowledge of DBT from pre- to post-training (*d* = 1.53)* and to follow-up (ES = 1.56)*; no significant differences between post-training and follow-up. **Attitudes towards use of DBT strategies in clinical practice (Level 2):** -Large improvement from pre- to post-training (*d* = 1.76*) and to follow-up (*d* = 1.17)*; no significant differences between attitudes at post-training and at follow-up. **Perceived Behavioural Control (PBC; Level 2):** - Large improvement from pre- to post-training (*d* = 1.36*) and to follow-up (*d* = 1.68)*; no significant PBC differences between post-training and follow-up. **Intentions to use DBT (Level 2):** -Large improvement in intentions from pre- to post-training (*d* = 0.80*); no significant differences between intentions at pre-training and follow-up, or post-training and follow-up. **Self reported DBT behaviours (Level 3):** Behaviours increased from pretraining to 6-month follow-up (*d* = 0.61)*.
Harned et al. (2021) [46]	U.S. and U.K., range of services *DBT-Prolonged Exposure (DBT-PE)*	Observational repeated measures	240 clinicians; 82.7% cisgender woman, 0.4% transgender; 92% “Caucasian”; 85% licensed professionals, 8.8%, post-graduate trainee, 5.4% graduate trainees; 93.7% very familiar with DBT; 32.8% very familiar with imaginal exposure for PTSD, 25.5% very familiar with in vivo exposure for PTSD.	DBT-PE workshops: −2 days (Harned) −4 days (Harder and co-trainer; same content but more depth and practice). No post workshop consultation or ongoing support provided.	Pre vs post-training vs 3-month vs 6-month follow-up 2-day workshop vs 4-day workshop	**Concerns About Worsening scale (Level 2):** -Decreased from pretraining to the 6-month follow-up*. -On average, clinicians who attended the 2-day workshop had more concerns than 4-day workshop*; the rate of change did not differ by workshop type. **Perceived treatment credibility for treating PTSD in high-risk BPD patients (Credibility Scale; Level 2):** -Increased from pre-training to 6-month follow-up*. −2-day attendees initially rated the treatment as less credible than 4-day attendees*; 2-day attendees showed a greater increase in credibility over time than 4-day attendees*. **Self-efficacy in using exposure to treat PTSD (including in patients with comorbid BPD; Level 2):** -Increased from pre-training to 6-month follow-up*. −4-day attendees initially reported greater comfort using exposure than 2-day attendees*; the rate of change in self-efficacy over time did not significantly differ between the groups. **Actual DBT-PE use (n = 183; Level 3):** −53.5% reported using DBT-PE 6 months after training, delivering the protocol to 241 patients (*M* = 2.5; *SD* = 2.1). -Average satisfaction with using DBT-PE was high; 100% of adopting clinicians reported intending to continue using DBT-PE in their practice. -The strongest predictors of DBT-PE use during follow-up were higher post-training self-efficacy with DBT-PE*, higher perceived credibility of DBT-PE post-training*. -Workshop type, prior exposure experience, and pre-training DBT self-efficacy were not significant predictors. **Barriers to use (Level 3):** Reported barriers pre-training: lack of training (58.3% [7.3% 3 months, 15.1% 6 months]), concerns patients would decompensate (36.7% [2.4% 3 months, 2.7% 6 months]), not prepared for potential side effects (20.8% [2.4% 3 months, 4.1% 6 months]), lack of confidence in treatment (20.0% [1.2% 3 months, 1.4% 6 months]). −3-month follow-up most common reason: patients not appropriate for DBT-PE (53.7%). Other concerns: Time (7.3%), patient disinterest (15.9%). −6-month follow-up: patients not appropriate (47.9%), time concerns (12.3%), patient disinterest (13.7%).
Perseius et al. (2007) [47]	Sweden, adult and child psychiatry clinics	Mixed-methods, observational repeated measures	22 therapists working with women with BPD; 86.4% female; 36.4% registered nurses, 36.4% mental care assistants, 9.1% physicians, 13.6% psychologists, 4.5% occupational therapists.	6 months education followed by 18 months of implementation and supervised treatment. Total education (provided by experienced DBT therapists): 110 hours of theory, 51 workshop hours of method and mindfulness training, and 153 hours of supervision in teams or individually.	Pre vs post-training: baseline and 6, 12 and 18 months in the treatment phase.	**Burnout (MBI; Level 3)** -Overall burnout showed a brief mid-point rise, but no significant or lasting change.
Carmel et al. (2014) [48]	U.S., public behavioural health system	Observational repeated measures	34 mental health practitioners and substance abuse counsellors; 88% female, mean of 1.2 years (*SD* = 3.16) DBT experience.	Comprehensive DBT training (expert DBT trainer): 10 days (80 hours), over period of 13 months (noted as different to standard DBT-ITM model).	Pre training vs post-training (13 months) *N = 9*	**Burnout (CBI; Level 3):** -Mean burnout reduced from pre- to post-training (*d* = 0.76)*.
Harned et al. (2021) [49]	U.S., one adult outpatient DBT program, two residential DBT programmes (one adult, one adolescent), one Assertive Community Treatment program DBT-PE	Observational study with external benchmark comparison	17 therapists providing DBT; 58.8% female; 76.5% White, 17.6% African American, 5.9% multiracial; 64.7% master’s-level social workers, 11.8% doctoral-level psychologists; mean 15.5 months providing DBT at agency (*SD* = 14.6); average DBT caseload 3.7 clients (*SD* = 3.3, range 0–13).	Provided 4 days DBT-PE workshop + 16 months ongoing support (Harned and colleagues; 32 hours bimonthly team consult). Previous training: M days workshop training in DBT = 12.5 (*SD* = 8.4); 17.6% (*n* = 3) had previously attended DBT-PE workshop and used DBT-PE. Benchmark comparison: All therapists had DBT-ITM. Two were certified PE therapists and supervisors; therapists not certified in PE attended a 1-day DBT-PE workshop and received supervision from a certified PE supervisor.	Therapist adherence vs adherence benchmark (efficacy trial benchmark)	**DBT-PE adherence (Level 3):** -On average, DBT-PE sessions were delivered with good to excellent adherence (*M* = 2.70, *SD* = 0.60). Of the 48 coded DBT-PE sessions, 46 (95.8%) were adherent. -Compared to adherence benchmark (*M* = 2.90, *SD* = 0.20), community clinicals delivered DBT-PE with comparable adherence. **DBT Adherence (DBT ACS; Level 3):** -On average, clinicians delivered DBT below adherence (*M* = 3.90, *SD* = 0.20,); 48.3% sessions were below adherence; 51.7% were adherent. -Compared to adherence benchmark (*M* = 4.10, *SD* = 0.20), community clinicians were less adherent to DBT (*d* = 0.90)*.
Harned et al. (2024) [50]	U.S., range of mental health settings	Secondary data analysis	63 therapists in total: **Study 1 (n = 13):** Therapists delivering DBT, regardless of training experience, in an effectiveness trial for DBT-PE; 61.5% female; 92.3% Non-Hispanic White; 15.4% psychologists, 30.8% social workers, 53.8% counsellors; mean 1.9 years DBT experience (*SD* = 1). **Study 2 (n = 50):** Therapists delivering individual DBT with some DBT training; 84% female; 84% Non-Hispanic White, 10% Hispanic White, 6% Asian; 24% psychologists, 34% social workers, 36% mental health counsellors; mean 6 years DBT experience (*SD* = 3.8).	**Study 1:** May have had previous training. As part of the study, attended a 4-day workshop in DBT-PE followed by 16 months (32 hours) of bimonthly team consultation with an expert. Previous training: -Linehan 1993 manual: 92.3% -Linehan 2015 skills manual: 53.8% -Supervision with agency: 61.5% -Supervision with external expert: 100% -DBT online training course: 15.4% -DBT workshop: 100% -Workshop days: *M* = 13.7 (*SD* = 7.8) **Study 2:** No training as part of the study. Given ACI manual and measure to review. All therapists had completed some training, defined as at least one of the following: 2+ days of workshops, a graduate level course in DBT, 6+ hours of online training, or reading any of Linehan’s DBT manuals. Previous training: -Linehan 1993 manual: 100% -Linehan 2015 skills manual: 98% -Supervision with agency: 62% -Supervision with external expert: 60% -DBT online training course: 50% -DBT workshop: 98% -Workshop days: *M* = 18.9 (*SD* = 16.4)	Study 1 vs Study 2	**DBT adherence (DBT ACS; Level 3):** -No differences between the two samples in average global adherence or the rate of adherent sessions. -Therapists delivered DBT individual therapy below adherence on average (*M* = 3.96, *SD* = 0.18; cut-off for adherence = 4.00). -Overall, 54.4% of sessions were adherent and 45.6% of sessions were non-adherent.
DiGiorgio et al. (2010) [51]	U.S. range of settings	Cross-sectional	129 therapists who had completed at least one Behavioral Tech training and conducted DBT with at least one client; 79% female; 95% White; 32.3% clinical psychologists, 30% social workers, 9.4% counselling psychologists.	DBT-ITM (Behavioral Tech): 39.4%. No DBT-ITM (Behavioral Tech): -Two-day skills training: 63.8% -Two-day individual psychotherapy training: 57.5% -Five-day mindfulness workshop: 6.3% -Five-day advanced DBT individual training: 4.7%.	DBT-ITM vs No DBT-ITM	**Self reported DBT Adherence (Level 3):** -No significant difference between therapists who had attended DBT-ITM vs other trainings. **Self-Reported DBT Components in Individual Therapy (Level 3):** -DBT-ITM therapists more frequently used skills groups (*d* = 0.41)*, consultation teams (*d* = 0.34)*, phone consultations (*d* = 0.30)*, diary cards/homework (77.5% vs. 55.4%)*, and target hierarchy (55% vs. 21.4%)* than non-DBT-ITM therapists. -Non-DBT-ITM therapists more often reviewed/applied skills (75% vs. 47.5%)*. -DBT-ITM therapists used interpersonal effectiveness skills in non-DBT sessions (39.5% vs. 15.5%)*, modified homework sheets more (33.3% vs. 13.8%)*, made fewer consultation team changes (5.1% vs. 24.1%)*; more DBT-ITM therapists (25% vs. 7.35%)* reported positive outcomes from modifications. **Integration of DBT with other approaches (Level 3):** -More DBT-ITM therapists reported using humanistic approaches in combination with DBT (25.0%) than non DBT-ITM therapists (6.5%)*. -More non DBT-ITM therapists reported integrating at least one DBT skills module into their usual therapeutic approach, compared to DBT-ITM therapists (32.6% vs 5.9%)*.
Trupin et al. (2002) [52]	U.S., Juvenile Rehabilitation Administration (JRA) facility	Retrospective quasi-experimental	Staff: worked in either mental health cottage unit or general population unit. Clients: 61 adolescent incarcerated females: −23 from mental heal cottage unit (MHC) −23 General Population Cottage with DBT (GPCD) −15 General Population Comparison Cottage (GPCC).	Four staff from MHC received extensive DBT training (Linehan and colleagues; 80 hours). The remaining staff from MHC, and staff from GPCD received DBT introductory training (Linehan’s associates; 16 hours + weekly consultation for one year).	Time series over DBT year on different units DBT units vs year prior matched comparisons	**Staff punitive actions (Level 3):** -No within-year change in staff punitive actions during DBT implementation on MHC; fewer punitive actions overall during the DBT year compared to year prior*. -Increase in staff punitive actions on GPCD during the DBT implementation (*R*^*2*^ = 0.74)*. **Client behaviour problems (Level 4):** -Reduction for youth on MHC (*R*^*2*^ = 0.55)* over DBT year, but not GCPD. -No change in youth behaviour between the DBT and non-DBT year on MHC. **Client Risk Assessment scores (Level 4):** -No significant difference in risk score changes between MHC, GPCD and year prior matched comparisons; MHC and GPCD groups showed within-group improvements over time*.
Tebbett-Mock et al. (2020) [53]	U.S., adolescent inpatient unit, private psychiatric hospital	Retrospective quasi-experimental	Staff: 9 DBT clinicians, 35 ward staff. Clients: 801 inpatient adolescents (age 12–17).	DBT clinicians: DBT-ITM (Behavioral Tech). Ward staff: 3-hour didactic training. Psychology trainees: 10-hour didactic series.	Pre-training TAU vs post-training DBT implementation	**Client outcomes (Level 4):** -Fewer constant observation (CO) hours post-training (*r* = 0.09)* -Fewer suicide attempts post-training (*r* = 0.10)* -Fewer self-injury incidents post-training (*r* = 0.07)* -Fewer restraints post-training (*r* = 0.09)* -Fewer hospital days post-training (*r* = 0.10)* **Cost savings (Level 4):** -$251K saved in CO staff time over 8 months post-training
Tebbett-Mock et al. (2021) [54] Note: follow-up to 2020)	U.S., adolescent inpatient unit, private psychiatric hospital	Retrospective quasi-experimental, 3 cohort comparison	Staff: 9 DBT clinicians, 31 ward staff. Clients: 1194 adolescents (age 12–17) in 3 cohorts (TAU, DBT Group 1, DBT Group 2).	Group 1 (2020 study): DBT-ITM training for clinicians plus 3-hour didactic training for ward staff. Group 2: two new DBT team members completed foundation training (Behavorial Tech); seven new ward staff received no DBT training.	Group 1 vs Group 2 Group 2 vs TAU	**Client outcomes (Level 4):** -Group 1 had significantly fewer suicide attempts (*E*^*2*^ = 0.01)* and self-injury (*E*^*2*^ = 0.05)* vs Group 2. -No significant difference between Group 1 and 2 for CO hours, restraints, number of days hospitalised. -Group 2 had more self-injury vs TAU (*E*^*2*^ = 0.05)*, despite fewer CO hours (*E*^*2*^ = 0.01)* -No significant differences were found for suicide attempts, restraints, number of days hospitalised.
Pasieczny & Connor (2011) [55]	Australia, routine public mental health settings	Quasi-experimental	Staff: 18 DBT therapists (range of professions). Clients: 90 adults who met criteria for BPD.	All DBT therapists completed 4 days of basic DBT training (local experienced trainers). Four DBT therapists completed 10 days intensive training (Behavioral Tech). No specialist supervision.	Clients of basic trained staff vs clients of intensive trained staff	**Client outcomes (Level 4):** - DBT-ITM clients showed greater reductions in suicide attempts (*d* = 1.59 vs 0.98)* and self-injury (*d* = 0.95 vs 0.48)* than those with basic-trained therapists. -No group differences were found for anxiety, distress, or service use.
King et al. (2018) [56]	U.K., teams trained by biDBT	Mixed methods; retrospective cohort study using survival analysis methods	68 DBT teams; 91% active, 9% inactive, from a range of services (46% ACMHT, 19% CAMHS).	DBT-ITM (British Isles DBT Training)	Early (before April 2007) vs late (after April 2007) cohort Trained off-site vs onsite	**Survivability (Level 4):** -Late adopters were more likely to stay active longer than early adopters (*d* = −0.39)*; programme closures peaked in Year 2, regardless of when they were started. -DBT programmes trained off-site had better sustainability than those trained onsite (*d* = 0.73)*; programme closures peaked in Year 2 for onsite training and Year 3 for off-site training.

*Note.* ABPDQ = The Attitudes toward BPD Questionnaire [57]; BAQ = Behavioral Anticipation and Confidence Questionnaire [39]; BPD = Borderline Personality Disorder; CBI = Copenhagen Burnout Inventory [58]; DBT = Dialectical Behaviour Therapy; DBT-A = Dialectical Behaviour Therapy for Adolescents; DBT ACS = DBT Adherence Coding Scale [59]; DBT-ITM = The DBT Intensive Training™ Model; DBT-PE = Dialectical Behaviour Therapy Prolonged Exposure; MBI = Maslach Burnout Inventory [60]; PTSD = Post-Traumatic Stress Disorder; RCT = Randomised Controlled Trial; TAU = Treatment As Usual; U.K. = United Kingdom; U.S. = United States

Only comparators relevant to the review are reported. Where studies are mixed-methods, only quantitative data are reported. Effect sizes presented in chronological order. Scales are reported where validated measures were used. Where there is no reported scale, a study-designed measure was used

*p < .05

Study settings also showed considerable variation. Fifteen were conducted in the United States, two were in the United Kingdom, and one across both countries. The remaining five were conducted in Singapore, Sweden, Australia, Canada, and across Spain and Latin America. Services varied widely and included both inpatient and community settings, serving diverse populations such as individuals with mental health or substance use issues, veterans, people with learning disabilities, adolescents, and those in juvenile rehabilitation.

Across the included studies, 1,411 staff and 1,345 clients participated. Staff sample sizes ranged from 9 to 240 (*n* = 21; *M* = 67.19, *SD* = 64.20), while client sample sizes ranged from 61 to 1,194 (*n* = 3; *M* = 448.33, *SD* = 645.93). One study included 68 DBT teams. Most staff participants were mental health therapists, although three studies focused specifically on ward staff, such as health care assistants and nurses. Client populations included adolescent incarcerated females, inpatient adolescents and adults meeting criteria for BPD.

Descriptions of DBT training varied widely, though most studies described using both active and passive teaching styles. Five studies reported using the DBT Intensive Training Model (DBT-ITM), five described broader training-implementation initiatives, and four focused solely on teaching DBT skills. Other training formats included foundational or basic DBT training, partial DBT-ITM, and a DBT awareness training. Training durations ranged from 2.5-hour workshops to 18-month programmes. While most studies reported training in standard DBT, two focused on the DBT Prolonged Exposure protocol (DBT-PE), and one used a DBT skills programme tailored for people with learning disabilities (“I Can Feel Good”).

Training delivery formats also differed: seven studies used only in-person training, seven included training plus implementation support, and three used online formats. Three studies involved multiple trainings, and three RCTs compared different training formats. Eleven studies reported trainings delivered by Behavioral Tech Institute, which is an established DBT training company in the U.S. Nine studies reported using DBT experts, and others employed training from groups such as British Isles DBT Training, DBT Iberoamérica, or in-service psychologists.

Studies utilised many different comparators. Nine studies only had a pre–post comparator. Other comparators included different training formats, settings, lengths and periods, cohort comparisons, comparisons to benchmarks, comparisons between studies, and control group comparisons.

**Fig. 2:**
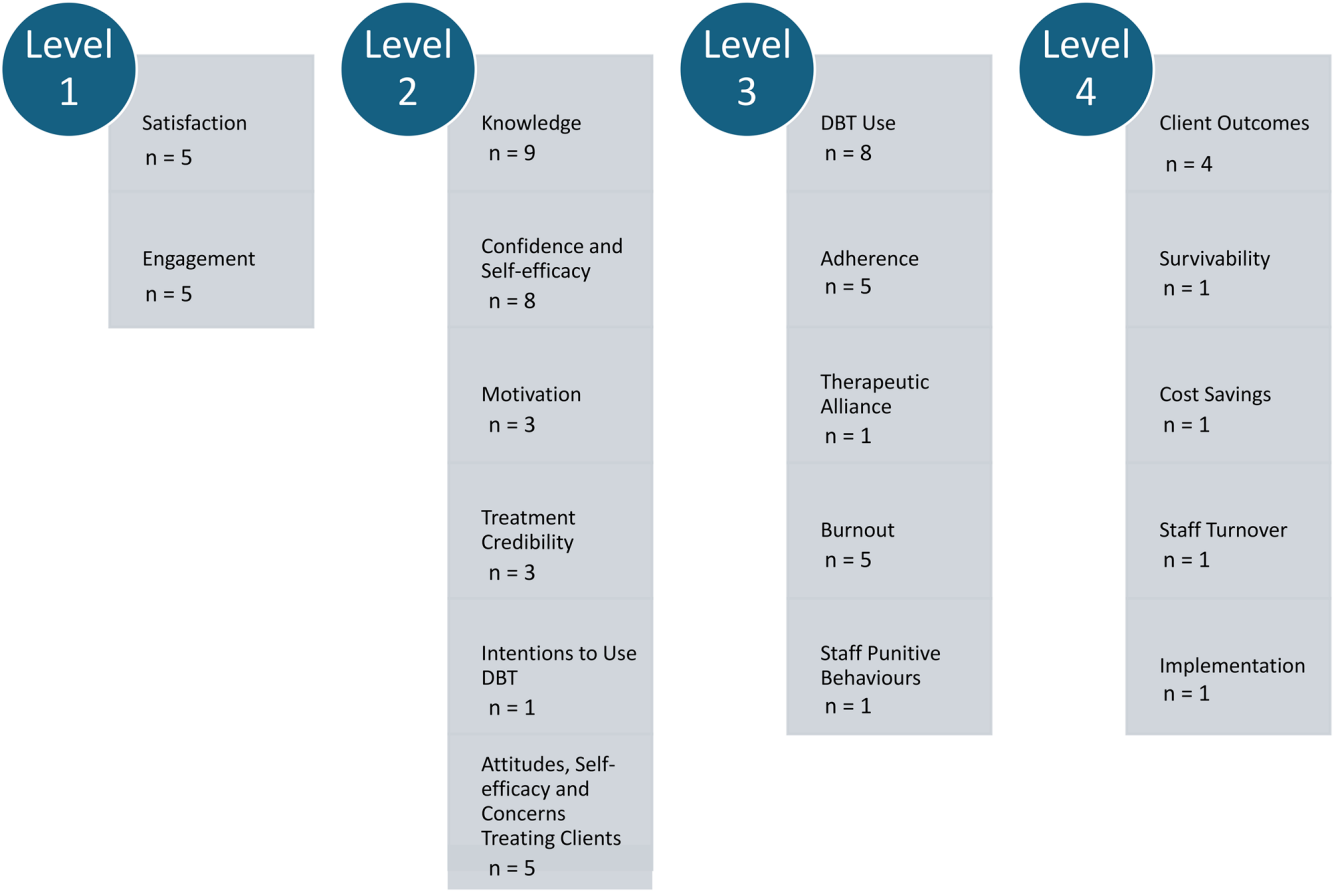
Outcomes reported across Kirkpatrick levels within studies

Outcomes studied and reported were also highly variable (Fig. 2). Six studies reported Level 1 outcomes (reaction), thirteen studies reported Level 2 outcomes (learning), sixteen studies reported Level 3 outcomes (behaviour), and six studies reported Level 4 outcomes (results).

Most studies were assessed as having moderate risk of bias (Supplementary File 5). Certainty of evidence was assessed for six priority outcomes, including knowledge, confidence/self-efficacy, adherence, use of DBT in clinical practice, implementation outcomes, and client suicide and self-harm outcomes. To evaluate the overall effectiveness of DBT training, GRADE assessments were conducted separately for different comparisons: pre–post, and DBT training versus control (which encompassed different training modalities, supporting exploration of training components). Additionally, outcomes from four studies comparing more intensive to less intensive DBT trainings were graded to address the second research question regarding which training components (e.g., duration, format) are associated with better outcomes (see Table 2 for a summary and Supplementary File 6 for the detailed assessment).

**Table 2:** GRADE assessment summary

Outcome	Comparison	No. of Studies (Participants)	Effect Estimate	Certainty of Evidence
DBT knowledge acquisition	Pre–post	5 (125)	Improvement post-training; large effect in one study (*d* = 1.53–1.56)	⨁◯◯◯ Very low
	DBT training vs no training	1 (109)	Trained group scored higher	⨁◯◯◯ Very low
	DBT training vs manual	3 (454)	Medium effects for DBT skills training compared to manual (*d* = 0.36–0.62)	⨁⨁◯◯ Low
	Intensive vs. non-intensive	1 (109)	Higher scores for intensive training	⨁◯◯◯ Very low
Confidence/self-efficacy	Pre–post	5 (575)	Consistent improvement; one study large effect (*d* = 1.36–1.68)	⨁◯◯◯ Very low
	DBT vs. Manual	3 (454)	Mixed short-term effects only (*d* = 0.54–0.68)	⨁⨁◯◯ Low
	DBT vs. e-Control	1 (89)	e-DBT outperformed control (*d* = 0.76–0.83)	⨁⨁⨁◯ Moderate
Use of DBT in practice	Pre–post	3 (91)	Inconsistent; small–moderate effects (*d* = 0.30–0.61)	⨁◯◯◯ Very low
	DBT vs. manual	3 (454)	Inconsistent effects	⨁◯◯◯ Very low
	DBT vs. e-control	1 (89)	DBT training more effective	⨁⨁⨁◯ Moderate
	Intensive vs. non-intensive	1 (129)	More DBT use in intensive group (*d* = 0.30–0.41)	⨁◯◯◯ Very low
	DBT vs. manual	2 (322)	No significant difference	⨁⨁⨁◯ Moderate
	Intensive vs. non-intensive	1 (129)	No significant difference	⨁◯◯◯ Very low
Implementation outcomes	Pre–post	1 (61)	Significant increase in use (esp. mindfulness)	⨁◯◯◯ Very low
Client suicide/self-injury	Pre–post	2 (1,194)	Mixed; small effects (*r* = 0.07–0.10; ε^2^ = 0.05)	⨁◯◯◯ Very low
	Intensive vs. non-intensive	2 (1,194)	Lower risk with full training; small effects	⨁⨁◯◯ Low

## Results of syntheses

### Level One: Reaction

**Satisfaction.** Five studies assessed satisfaction using post-training self-report measures following DBT skills-focused trainings delivered via workshops, online modules, manuals, or blended formats.

*Randomised Controlled Trials (n = 3).* Three RCTs [39–41] compared instructor-led workshops, e-learning, and manual-based learning. Instructor-led and online formats were consistently rated more favourably than manual-based approaches (*d* = 0.65–1.55).

*Descriptive Surveys (n = 2).* Descriptive post-training surveys [30, 35] reported high satisfaction. Kauth et al. [30] found that 88% of participants were satisfied with online modules and 85% with facilitation calls; however, 73% reported difficulty completing modules during work hours and 72% found the accompanying manual unhelpful. Ashworth et al. [35] reported high mean satisfaction ratings for training relevance, usefulness, and facilitator quality following an in-person ward staff training.

Overall, findings suggest a preference for instructor-led and online training over manual-based self-study. However, heterogeneous measures, reliance on self-report, and variation in study quality limit cross-study comparability and confidence.

**Engagement**. Five studies assessed engagement using training completion rates, attendance, and time spent in training.

*Randomised Controlled Trials (n = 3).* Studies [39–41] had mixed findings, suggesting that interactivity may influence engagement. Dimeff et al. [39] found no significant differences in completion rates across instructor-led, e-learning, and manual conditions, whereas the 2015 study [41] reported higher completion rates for instructor-led workshops, possibly due to delays and reduced interactivity in the e-learning format. Dimeff et al. [40] found that participants completing e-learning spent significantly more time in training than those in manual (*d* = 2.11) and e-control conditions (*d* = 1.79), though these findings were obtained in controlled research settings and may not generalise to routine training.

*Repeated-Measures Studies (n = 1).* McCay et al. [34] reported mixed engagement with a multi-component training (e-learning, webinars, reading). Self-directed e-learning had high attendance when embedded within peer consultation meetings, while attendance at expert-led webinars was low, reportedly due to technical issues and organisational demands.

*Descriptive Surveys (n = 1).* Kauth et al. [30] found that 74% of participants attended all monthly facilitation calls following web-based training, indicating perceived value of ongoing support.

Overall, findings suggest that engagement may be shaped more by organisational and contextual factors than by training format alone. Structured facilitation and interactivity appear particularly beneficial in sustaining engagement with e-learning.

### Level Two: Learning

**Knowledge.** Nine studies assessed knowledge acquisition, eight used direct assessments (e.g., DBT Skills Knowledge and Application Test [39]), while Ashworth et al. [35] used a Likert-style self-report.

*Randomised Controlled Trials (n = 3).* Dimeff et al. [39–41] found all training conditions improved on knowledge test scores; however, e-learning led to more knowledge gains than instructor-led or manual formats (*d* = 0.36–0.62).

*Repeated-Measures Studies (n = 5).* Five studies used repeated measures to evaluate knowledge gained from varied training formats: multi-component implementation initiatives [34, 37], five-day comprehensive training [45], 12-hours skills trainings [42], and a one-day “I can feel good” training [35]. Only McCay et al. [34] utilised online methods. All reported statistically significant improvements in knowledge post-training (*d* = 1.53–1.56) [45], though sample sizes were small and certainty of evidence was very low due to risk of bias.

*Cross-Sectional Studies (n = 1).* Hawkins and Sinha [36] found that therapists with more extensive DBT training (e.g., workshops, consultation, study groups) scored significantly higher on knowledge assessments than those with less exposure. Reading DBT materials (*r* = 0.59), peer consultation (*r* = 0.63), and study groups (*r* = 0.65) were strong predictors. Combining formats also predicted higher scores. However, moderate risk of bias and lack of standardised measurement resulted in very low certainty of evidence.

Overall, varied DBT trainings are associated with improved knowledge. Two studies [36, 37] reported education level influenced scores, while behaviour therapy background did not [36]. E-learning may offer additive benefits, but limitations warrant replication to strengthen confidence.

**Confidence, Self-Efficacy, and Motivation.** Eight studies measured participants’ perceived behavioural control, confidence, self-efficacy, or motivation to apply DBT strategies. These outcomes were frequently measured together, although some studies assessed them separately. Five studies used The Behavioural Anticipation and Confidence Questionnaire (BAQ) [34, 39, 41, 43]. Other studies used study-designed measures.

*Randomised Controlled Trials (n = 3).* Dimeff et al. [40, 41] assessed self-efficacy and motivation separately, while Dimeff et al. [39] measured them together. In 2009, e-learning and instructor-led training showed a short-term BAQ improvement compared to reading a manual (*d* = 0.68, 0.54), though this was not sustained or consistent across studies measuring self-efficacy and motivation separately [40, 41]. In the 2015 study [41], motivation decreased over time in all groups; instructor-led training had the highest post-training motivation. These inconsistencies suggest measurement method may influence observed outcomes.

*Repeated-Measures Studies (n = 5).* Studies using repeated measures designs evaluated various training formats ranging in intensity, from DBT-ITM to one-day workshops [34, 35, 43, 45, 46]. All reported significant improvements in confidence and/or self-efficacy post-training (*d* = 1.36–1.68) [45], however, risk of bias and other study limitations led to a very low certainty of evidence. Harned et al. [46] found that self-efficacy change did not differ by DBT-PE workshop length (two or four-days), suggesting that duration may be less influential than anticipated, however, this may reflect participants’ prior familiarity with DBT. Confidence was moderately correlated with DBT implementation (*r* = 0.33), and self-efficacy in managing suicide risk (*r* = 0.43) [43], suggesting that gains in confidence may generalise to other training outcomes. Motivation outcomes were mixed: Ashworth et al. [35] reported gains, while Navarro-Haro et al. [43] found no significant increases in motivation following DBT-ITM, though this may represent a ceiling effect. McCay et al. [34] observed a non-linear trajectory for combined motivation and self-efficacy: increased post-training, a dip at 4–6 months, and a rise at 12–16 months.

Overall, participants often reported improved self-efficacy and confidence post-training, though methodological limitations, variability in measurement, and inconsistent results mean it is unclear how much change is attributable to DBT training.

**Treatment Credibility.** Three repeated-measures studies found consistent pre–post improvements, though formats and measures varied [38, 45, 46]. Two studies [38, 46] found greatest increases among attendees with low initial confidence. Bender et al. [45] reported large effect sizes (d = 1.17, 1.76). However, absence of control groups limits internal validity and may reflect demand characteristics or regression to the mean.

**Intentions to Use DBT.** Only one repeated-measures study assessed intentions to use DBT in clinical practice [45] and found a significant increase from pre- to post-training (*d* = 0.8), though this was not sustained at six-month follow-up. While promising, the small sample size (*n* = 16) and absence of a control group limit confidence.

**Attitudes Towards Clients with BPD.** Three studies evaluated attitudes towards clients with BPD following DBT-ITM [38], five-day DBT-ITM part one [44] and a 12-hour skills training [42]. Herschell et al. [38] used a study designed measure, whereas Tan et al. [44] and Haynos et al. [42] used the Attitudes Towards BPD Questionnaire [57].

*Controlled Repeated-Measures Designs (n = 1).* Tan et al. [44] found part one of DBT-ITM reduced negative clinician stigma (Eta squared [*η*^*2*^_*p*_] = 0.30), though the small sample size (*n* = 14) may overestimate effects.

*Repeated-Measures Designs (n = 2).* In two studies, DBT training was associated with reduced personality disorder stigma [38, 42], with the greatest improvements among those with more negative baseline attitudes (*r* = −0.46) [38], though this may reflect regression to the mean.

Overall, DBT training may reduce clinician bias, but evidence is limited to intensive formats and inconsistent measurement reduces comparability.

**Self-Efficacy and Concerns Treating Suicidal Clients.** In two repeated measures studies, Navarro-Haro et al. [43] found Clinician Self-Efficacy in Managing and Assessing Suicide Risk [61] scores increased significantly post-DBT-ITM, while Concerns About Treating Suicidal Patients [61] remained unchanged; however, subscale analysis showed reductions in Training and Competence concerns. This suggests DBT-ITM may be associated with enhanced perceived competence and reduced training-related concerns. Notably, participants who did not complete the post-training assessment had significantly higher baseline self-efficacy and lower concerns than pre–post completers, suggesting that the analytic sample may overestimate the training impact.

**Concerns About Exposure Worsening Client Outcomes.** In a repeated measures study comparing workshop lengths, Harned et al. [46] found that clinician concerns about DBT-PE worsening client outcomes significantly declined from pre-training to six-month follow-up across workshops, thus, training was associated with reduced clinician apprehension about using DBT-PE protocols, regardless of workshop length. However, the absence of a control group limits causal interpretation.

### Level 3: Behaviour

**DBT Use.** Eight studies used self-report measures to assess whether DBT training influenced participants application of DBT strategies.

*Randomised Controlled Trials (n = 3).* Studies comparing different training formats found mixed results. Two RCTs by Dimeff et al. [39, 41] found no significant differences between instructor-led workshops, e-learning, and reading on DBT application at 90-day follow-up. This suggests workshops and e-learning are no more beneficial than reading a manual; though, strategy use did increase over time in the 2015 study. Contrastingly, Dimeff et al. [40] found that e-DBT training produced more consistent DBT use than manual or control conditions. These mixed findings may reflect differences in the specific skills measured, or participant engagement with the training formats, demonstrating the need for further research to clarify the impact of format, and what moderates the effect.

*Repeated-Measures Studies (n = 4).* Two uncontrolled studies evaluating comprehensive training [45] and DBT-ITM [38] reported moderate increases in DBT component use following training (*d* = 0.61) [45]. Notably, Herschell et al. [38] observed behaviour change was associated with improved attitudes towards BPD (*r* = 0.30) and confidence in DBT credibility (*r* = 0.45), suggesting behaviour change may be moderated by these outcomes. Conversely, Ashworth et al. [35] found no significant perceived behaviour change following a one-day training.

In another repeated-measures study, Harned et al. [46] reported that 53.5% of therapists used DBT-PE within six months post-workshop. Post-training self-efficacy and perceived credibility were the strongest predictors of use, while workshop length, prior experience, and baseline self-efficacy had no influence. Reported barriers to use at pre- and post-training shifted from intervention-focused (e.g., lack of training, safety concerns) to client-focused (e.g., perceived inappropriateness for DBT-PE).

*Cross-Sectional Studies (n = 1).* DiGiorgio et al. [51] found that therapists who received a 10-day intensive DBT workshop demonstrated greater use of core DBT components (skills groups, consultation, etc.; *d* range = 0.30–0.41), and strategies (diary cards, target hierarchies), than therapists who completed less intensive DBT trainings. No significant group differences emerged for strategies such as validation, problem solving, and teaching new skills, suggesting DBT training is primarily associated with increased use of techniques more specific to DBT. Findings cautiously suggest that more intensive training may promote DBT use; however, unaccounted factors such as baseline DBT context and training differences amongst the comparison group may have independently influenced outcomes.

Overall, findings suggest that comprehensive or intensive DBT training is associated with increased application of DBT, however, the certainty of evidence was very low. Some evidence suggests that credibility and confidence may mediate these effects, and contextual factors, such as organisational support, may moderate outcomes. High-quality research is needed to clarify training effects and contributing factors.

**Adherence.** Five studies assessed DBT adherence using various methodologies.

*Randomised Controlled Trials (n = 2).* Dimeff et al. [39, 41] used structured role-plays scored by trained raters and found significant improvements in DBT skill performance across all training modalities. Notably, role plays may not fully reflect real-world clinical performance and changes beyond 90 days were not measured.

*Cross-Sectional Studies (n = 1).* DiGiorgio et al. [51] operationalised adherence by subtracting the number of techniques therapists reported adapting from the number they reported using in a session, and found no differences between intensively and non-intensively trained therapists. This measure is methodologically weak as it relies on self-report, uses no standardised coding system, and inaccurately equates adaptation with reduced adherence, undermining the validity and interpretability of the findings.

*Observational Studies with External Benchmark Comparison (n = 1).* Harned et al. [49] used behavioural coding of real therapy sessions using gold-standard measures: the DBT-PE Adherence Measure [62] and the DBT Adherence Coding Scale (DBT ACS) [59]. High adherence was achieved for DBT-PE following a four-day DBT-PE training, comparable to an efficacy trial (*p* > 0.05). Conversely, despite substantial prior training and experience, general DBT adherence remained below the adherence threshold, significantly lower than the DBT-ITM-trained benchmark (Hedges’ *g* = 0.9) [49].

*Secondary Data Analysis (n = 1).* Using the same measures, Harned et al. [50] found no significant differences in DBT adherence between two samples with different training exposures. While 54.4% of sessions met the adherence threshold (≥4.00), the mean adherence score fell just below the cut-off (*M* = 3.96), likely reflecting low scores in non-adherent sessions; thus, prior training and experience did not predict adherence, highlighting the difficulty of achieving adherence in routine settings. Training effectiveness outcomes are lacking internal validity due to the lack of confounding variables considered and accounted for (e.g., experience, client characteristics, time since training).

Overall, while training is associated with improved adherence in short-term assessments, maintaining adherence in routine practice remains a significant challenge. More rigorous, longitudinal studies are needed to understand which training components promote sustained adherence.

**Burnout.** Five repeated measures studies found mixed results on the impact of training on therapist burnout. Four utilised the Copenhagen Burnout Inventory [58], while Perseius et al. [47] used the Maslach Burnout Inventory [60]. Only Tan et al. [44] had a control group.

Three studies evaluating comprehensive or intensive DBT training [43, 44, 47] found no significant effects for training on burnout levels. Perseius et al. [47] observed a temporary increase during training, possibly reflecting a tension around adopting new models. Conversely, Carmel et al. [48] reported a large post-training reduction (*d* = 0.76), though this is limited by a lack of control and small sample (*n* = 9).

Organisational and attitudinal factors may moderate burnout. Navarro-Haro et al. [43] found that burnout was positively associated with perceived structural and administrative barriers to implementation (*r* = 0.37). Haynos et al. [42] observed reductions in personal and work-related burnout following a 12-hour skills training for nurses, and found reductions in stigma toward BPD were strongly associated with burnout improvement (*r* = 0.62– 0.79), indicating a possible mediating role.

Together, findings suggest that while DBT training is sometimes associated with reduced burnout, these effects are not consistent. Self-report measures, small samples, and lack of control groups and long-term evaluation limit conclusions. Organisational climate, stigma, and structural barriers warrant further exploration as potential moderators.

**Therapeutic Alliance.** Only Tan et al. [44] examined the impact of training on therapeutic alliance in a controlled repeated-measures study. Using the Working Alliance Inventory–Short Revised Therapist scale [63], they found no effect for training on therapist-reported alliance. This highlights a notable gap in the literature.

**Staff Punitive Behaviours.** Trupin et al. [52] examined the impact of DBT training on staff punitive behaviours in multiple units in a juvenile rehabilitation facility. On the mental health unit, four staff received extensive DBT training (80 hours), while others received a 16-hour introductory training with weekly consultation. Staff in the general population unit only received the introductory training. No pre–post change was observed in the mental health unit, but significantly fewer punitive incidents occurred during the DBT year compared to the prior year. Conversely, punitive behaviours increased in the general population unit. Findings suggest that training intensity may influence staff behaviour, though limitations such as unreliable reporting of staff behaviour, and lack of adherence measures, limit interpretability.

### Level 4: Results

**Implementation.** Navarro-Haro et al. [43] evaluated the impact of DBT-ITM on DBT implementation in Spain and Latin America using part of the Program Elements of Treatment Questionnaire [64]. They found that DBT-ITM significantly increased the use of several DBT modalities (e.g., group skills, consultation teams, mindfulness), but not individual therapy. Implementation was negatively correlated with reported structural barriers (*r* = −0.33) and higher client caseloads (*r* = −0.33). While promising, certainty of evidence is very low and generalisability is limited due to reliance on a single study.

**Survivability.** King et al. [56] evaluated the long-term sustainability of DBT programmes in the UK using survival analysis on 68 teams who received DBT-ITM between 1995 and 2016. They found that DBT programmes trained off-site had greater longevity than on-site-trained teams *(d* = 0.73), and that survival improved after 2007 (*d* = −0.39). While the large sample strengthens the findings, potential confounders (e.g., staffing, setting), uneven follow-up data, and limited information on dropout reasons warrant caution before attributing sustainability outcomes solely to training location or timing.

**Staff Turnover.** Holbrook et al. [37] found that staff turnover decreased post-training in four of five programmes using the four-phase DBT training model, with one showing a statistically significant reduction. Notably, agency staff turnover slightly increased. However, internal validity may be limited by the lack of a control group and limited demographic detail.

**Cost Savings.** In a pre–post comparison, Tebbett-Mock et al. [53] reported $251,609 in cost savings from reduced staff observation time following DBT-ITM in an adolescent inpatient unit. This suggests that DBT training may be associated with improved resource efficiency; however, it remains unclear whether cost savings are directly attributable to training intensity or quality.

**Client Outcomes.** Four naturalistic studies assessed client outcomes following DBT training of varying intensities. While the findings suggest that more intensive DBT training may be associated with reduced acute risk behaviours, the certainty of evidence was rated very low, limiting causal inference.

*Suicide Attempts and Self-Injury.* Three studies that examined suicide attempts and self-injurious behaviours using quasi-experimental designs found improvements associated with greater training intensity.

Tebbett-Mock et al. [53] reported reductions in suicide attempts and self-injurious behaviours among inpatient adolescents following DBT-ITM implementation (*r* = 0.07). A follow-up study [54] found that clients treated by a fully trained team (therapists and ward staff) had significantly fewer suicide attempts (Epsilon squared [*ε*^*2*^ ] = 0.01) and self-injurious behaviours (*ε*^*2*^ = 0.05) than a partially trained team, while the latter had more self-injurious behaviours than treatment-as-usual (*ε*^*2*^ = 0.05). While this may suggest that training is associated with reductions in client suicide and self-injurious behaviours, effect sizes were small, and methodological concerns limit interpretation.

In an adult outpatient sample, Pasieczny and Connor [55] found significantly greater reductions in suicide attempts (*d* = 1.59 vs 0.98) and self-injurious behaviours (*d* = 0.95 vs 0.48) among clients treated by intensively trained therapists (10-day training) compared to those treated by basic-trained therapists (four-day training). These findings suggest a more robust link between training intensity and acute risk reduction. However, without adherence measures, it is unclear whether improved outcomes were due to enhanced DBT delivery or other factors, such as staff motivation or client characteristics.

*Behaviour Problems and Risk Scores.* Trupin et al. [52] found significant pre–post reductions in behaviour problems in the highly trained unit (*R*^*2*^ = 0.55); however, overall behaviour problems did not differ significantly from those in the prior year. The basic-trained unit observed no pre–post changes. Pre–post improvements in client risk scores were observed across all units, including the comparison group, but no significant between-group differences were found, suggesting no clear impact of DBT training.

*Restrictive Practices and Hospital Use.* Three studies reported resource use outcomes. While Tebbett-Mock et al. [53] reported small pre–post decreases in constant observation hours (*r* = 0.09), use of restraints (*r* = 0.09), and hospital days (*r* = 0.10) following DBT-ITM, no consistent differences in resource use were observed between clients treated by intensively trained versus non-intensively trained therapists or teams [54, 55].

*Symptom Outcomes.* Pasieczny and Connor [55] found no significant differences between 10-day and four-day-trained therapists on client-reported symptoms of depression (Beck Depression Inventory–II [65]), suicidal ideation (Beck Scale for Suicide Ideation [66]), anxiety (State-Trait Anxiety Inventory [67]), and general distress (Brief Symptom Inventory [68]). These findings suggest that DBT-ITM may be more strongly associated with reductions in acute risk behaviours than with improvements in broader psychological symptoms.

## Discussion

While previous research has reviewed training therapists in evidence-based practice (EBP) [69], this is the first review to specifically focus on the effectiveness of training therapists in DBT, a complex and multi-faceted therapy to deliver. DBT trainings, across a range of formats and intensities, were generally well received and associated with increased therapist knowledge in DBT and self-reported DBT use. Organisational and client outcomes showed tentative positive effects. Despite these encouraging findings, the overall certainty of the evidence was low, and methodological limitations constrain causal inference, particularly for therapist behaviour (Level 3) and client and organisational outcomes (Level 4).

There was a general trend towards more intensive trainings with implementation support being associated with improvements in knowledge acquisition, motivation, self-reported DBT use and improved client outcomes, compared to workshop or online-only training. This mirrors broader psychotherapy training research, which has consistently found that workshops alone rarely produce sustained behaviour change, whereas ongoing consultation, supervision, and supportive organisational contexts produce more consistent positive training outcomes [70–72]. Intensive trainings, such as DBT-ITM, are generally resource-intensive and difficult for routine clinical services to implement [73]. As a result, combining different training formats (e.g., reading, workshops, and consultation) may provide a more feasible alternative for services, although this still requires organisational support structures, such as supervision and consultation. Online training may also contribute to dissemination efforts, particularly when interactive and engaging, which aligns with research suggesting that active learning approaches are most effective for teaching clinical skills [74]. For example, Dimeff et al.’s RCTs [39–41] found that e-learning produced higher knowledge scores than workshops or manual reading, even when interactivity was reduced and workshops were rated more favourably for satisfaction. However, behavioural change did not differ across formats despite the recognised importance of role-play in skills acquisition [75]. Together, these findings suggest that while online or workshop-based formats may support knowledge acquisition, translating learning into sustained behaviour change likely requires ongoing consultation or supervision.

There is some evidence that therapists with a lower-level of previous education may have less knowledge gains than those with a higher educational background (e.g., doctoral-level psychologists). DBT is a comprehensive principle-based treatment and requires therapists to learn and apply multiple skills and strategies, while balancing multiple dialectics (e.g., acceptance and change) [1], thus, learning DBT is not straightforward. It may be important for services to support therapists with lower educational backgrounds, or specific learning needs, to access additional learning opportunities to support knowledge acquisition, such as self-directed learning, or peer consultation.

Although not typically a specific aim of DBT training, DBT training was associated with more positive attitudes toward working with clients with personality disorder diagnoses. This is particularly important as this client group experiences high levels of stigma [76]. A possible reason for this is that DBT training offers an explanation, through the biosocial theory [1], that can help professionals make sense of their experiences and provides solutions which reduces therapists’ fear of not knowing how to help. Some research has evaluated DBT training as an intervention for clinician stigma, finding that DBT-informed workshops can reduce stigma related to BPD; however, stigma towards BPD remained higher than for other mental health diagnoses [77, 78]. While Haynos et al. [42] found that burnout scores were positively correlated with BPD-related stigma, there was no robust evidence that DBT training reduces burnout; only two studies reported decreased burnout scores, both limited by small sample sizes and lack of control groups. These findings suggest that additional factors may moderate the relationship between clinician attitudes and burnout.

While self-reported change is an important outcome, many Level 2 and Level 3 measures relied on self-reported knowledge, self-efficacy, or DBT use. This is problematic given known discrepancies between self-report and observed behaviour or adherence [79]. Consequently, evidence of actual clinical skill acquisition following DBT training remains limited. This reflects a broader issue in therapist training research, where training outcomes often assume improvements in competence without directly measuring them [71, 80]. Evaluating adherence is therefore critical. Linehan [1] emphasised the importance of weekly consultation to support therapists in maintaining DBT fidelity, and higher therapist adherence has been associated with improved client outcomes [20]. However, few studies assessed adherence, and only two used standardised adherence measures. While these studies suggest DBT training may improve adherence, findings from Harned et al. [49, 50] highlight the difficulty of sustaining adherence in routine practice. Given evidence that therapists may require at least 1.5 years of DBT training to reach adherence thresholds [80]. Future studies should incorporate more objective and standardised measures, including in vivo assessments and validated adherence scales to strengthen external validity, and conduct long-term follow-up measurements (e.g., 18 months).

Overall, organisational outcomes were modest and lacked control groups, limiting the conclusions that can be drawn regarding the direct impact of DBT training on organisational change. Of the ten studies where training included an implementation focus, only four evaluated organisational or implementation outcomes. This is particularly disappointing given that DBT is a team treatment. Importantly, these studies found that DBT training may be associated with reduced staff turnover and have associated cost savings, which are often concerns for administrators [73]. As expected, DBT-ITM increased implementation of DBT modes; however, structural barriers, clinician burnout, and high caseloads negatively impacted implementation, aligning with previous research highlighting that multiple contextual factors influence the successful implementation of EBP [18, 71, 81, 82]. These findings suggest that while training is necessary, it is not sufficient on its own to ensure implementation, highlighting the need for ongoing organisational support and facilitative structures.

Client outcomes are the ultimate goal of any therapy training [71]. Disappointingly, only four studies evaluated client outcomes using a DBT training comparator. Three studies found that more intensive DBT training was associated with reductions in client suicidal and self-harm behaviour compared to less intensive training. While some effect sizes were small, this still represents meaningful clinical change for such a high-risk population. As these studies only assessed Level 4 outcomes, though, it is unclear how training influenced outcomes. For example, was it that DBT was delivered more adherently, or did higher intensity training improve motivation? Future studies should aim to evaluate outcomes across Levels 1–4, and identify correlations between outcomes.

## Limitations and future directions

A number of limitations were evident across the reviewed studies and reflect broader methodological flaws that have persisted in the training literature for decades [72, 79]. High attrition rates were reported in many studies yet were rarely explored in depth. Attrition may be influenced by training variables, organisational constraints, or implementation challenges; thus, future research should examine the reasons for dropout to better understand barriers to training completion and implementation.

Other limitations include inconsistency in the measurement tools used and the lack of long-term follow-up to determine whether training effects are maintained. These issues limit comparability across studies and reduce confidence in conclusions regarding training impact. Future studies should incorporate more objective and standardised outcome measures, including in vivo assessments and validated adherence scales, and conduct longer-term follow-up evaluations. Greater consistency in measurement across studies would also improve comparability, and future research should prioritise commonly used and validated measures of DBT training outcomes.

**Fig. 3:**
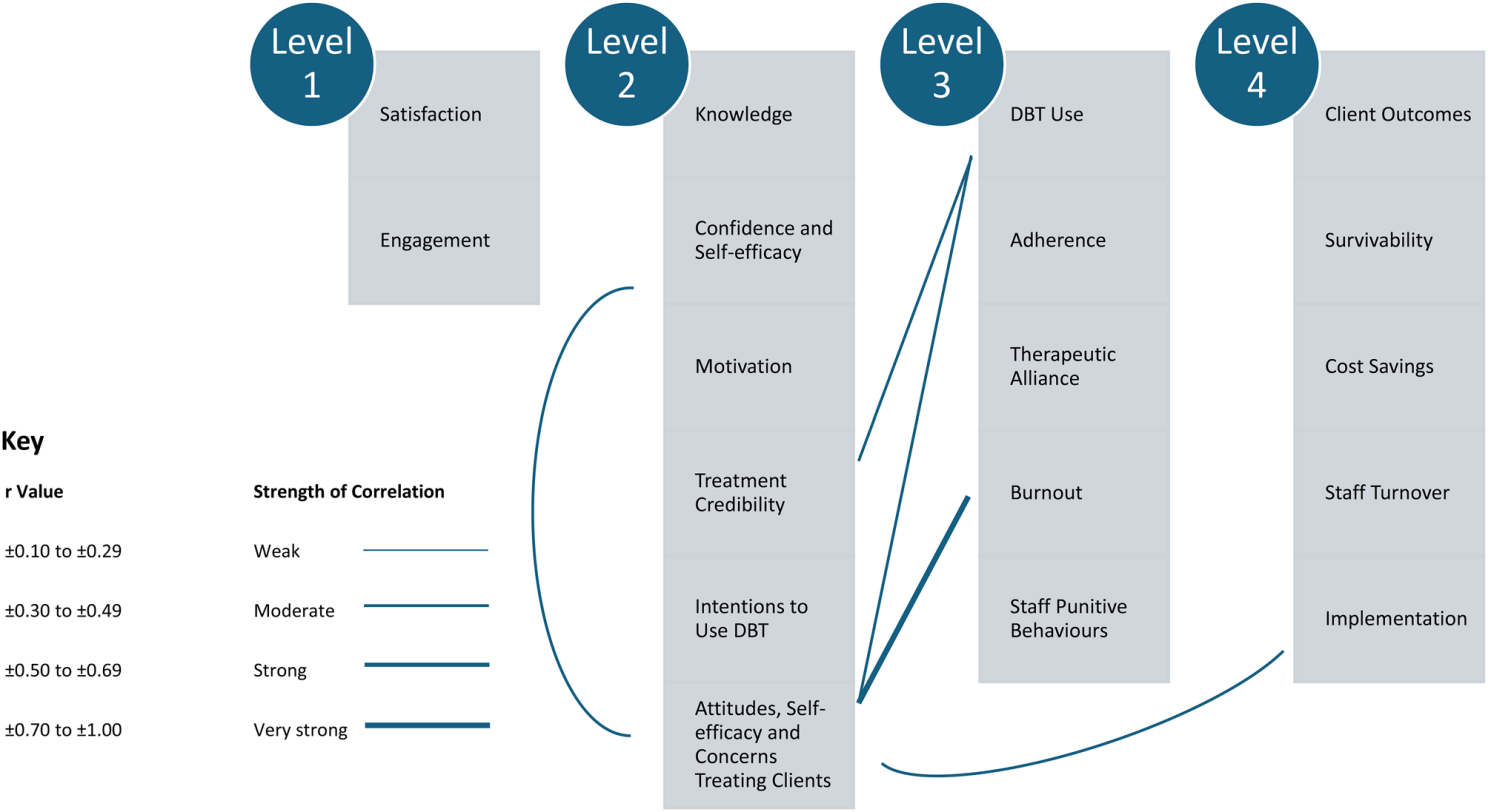
Outcomes obtained across levels with reported correlations within studies. Note: correlations were obtained from single studies only

Only a small number of studies examined relationships between outcomes across different levels (Fig. 3), highlighting a notable lack of empirical evidence linking training reactions (Level 1), learning (Level 2), therapist behaviour change (Level 3), and client and organisational outcomes (Level 4). Consequently, it remains unclear whether positive reactions to training or improvements in knowledge and skills translate into changes in clinical practice or improvements in client outcomes. Without examining these cross-level relationships, it is difficult to determine which aspects of training are most important for producing meaningful impact. This gap reflects a broader issue in psychotherapy training research: while knowledge gains are often reported, robust evidence linking these gains to skill acquisition and client outcomes remains limited [71, 83]. Future research should therefore explicitly analyse associations between outcome levels and investigate potential moderators, such as trainer enthusiasm, delivery format, and therapist characteristics, to better understand the mechanisms through which training leads to behavioural and client-level change.

This review used the Kirkpatrick Four Level Training Evaluation Model [21] as a framework to categorise training outcomes. While it offers a practical and widely accepted structure, it has recognised limitations, particularly its limited attention to contextual factors, such as organisational support and staff turnover, which are known barriers to implementation [84]. A recent adaption, the New World Kirkpatrick Model [85], aims to incorporate these broader influences. While this review did not utilise this model, as its complexity was less suited to a framework analysis and the expected outcomes, findings were interpreted with attention to broader contextual influences when these were reported. As the evidence base grows, future reviews may benefit from applying the New World model to systematically evaluate how organisational and contextual factors shape DBT training outcomes.

Finally, while every effort was made to capture all relevant literature, the review itself had some limitations. Studies were excluded if they were not peer reviewed, involved student samples, did not involve mental health professionals, lacked direct comparators, or did not report inferential statistics. Although these criteria ensured methodological rigour and focus, they may have led to publication bias and the exclusion of studies that could offer valuable insights, particularly regarding Level 3 and 4 outcomes, which are often embedded within broader implementation or effectiveness trials. Likewise, limiting inclusion to English-language publications may have resulted in the exclusion of relevant studies published in other languages. While two reviewers independently screened 10% of studies for inclusion and quality appraisal, data extraction and GRADE assessments were conducted by the lead researcher only, increasing the risk of subjectivity. Future reviews could benefit from broader inclusion criteria and more extensive independent review to further strengthen our understanding of the field.

## Conclusion

Overall, DBT training was often associated with positive outcomes across studies; however, causal inference remains limited by poor methodological rigour, a small number of studies, and moderate risk of bias. Mirroring Herschell et al. [83], more intensive training and implementation initiatives generally had the strongest outcomes, yet these approaches are resource-intensive and may limit the scalability of DBT. In response, more efficient, lower-cost methods such as online and blended training have been developed, showing promise, particularly for low-resource services. While online-based learning was generally well accepted, there is not enough evidence to suggest that it is sufficient without additional organisational support. While services often seek cost-effective training methods, investment in high quality training may be required to achieve positive outcomes. Future research should examine the training, organisational and contextual factors that shape training success.

## Electronic supplementary material

Below is the link to the electronic supplementary material.

Supplementary Material 1

Supplementary Material 2

Supplementary Material 3

Supplementary Material 4

Supplementary Material 5

Supplementary Material 6

## Data Availability

All data generated or analysed during this study are included in this published article and its appendices. Full search strategies for all databases are provided in Supplementary File 1.
