# Supplementary material for: The effectiveness of dialectical behaviour therapy training: a quantitative systematic review using Kirkpatrick’s four-level model
Source: Borderline Personal Disord Emot Dysregul. 2026 Apr 24;13:15. doi: 10.1186/s40479-026-00344-4 (PMC13244647; doi:10.1186/s40479-026-00344-4)
Supplement: Supplementary file 1 — Supplementary Material 1 [file 40479_2026_344_MOESM1_ESM.docx]

**Search Strategy**

**MEDLINE and CINAHL (via EBSCOhost)**

(MH "Education" OR MH "Training" OR "implement*" OR "train*" OR "workshop*" OR "teach*" OR "learn*" OR "student*" OR "develop*" OR "educat*" OR "course*" OR "disseminat*") XB Title and Abstract

AND (MH "Dialectical Behavior Therapy" OR "dialectical behaviour" OR "dialectical behavior" OR "dialectical behavioural" OR "dialectical behavioral") XB Title and Abstract

Search modes: Find all my search terms

Applied Limits: peer reviewed, English language

**Psycinfo (Proquest)**

(MH "Education" OR MH "Training" OR "implement*" OR "train*" OR "workshop*" OR "teach*" OR "learn*" OR "student*" OR "develop*" OR "educat*" OR "course*" OR "disseminat*") TIAB Document title & abstract

AND (MH "Dialectical Behavior Therapy" OR "dialectical behaviour" OR "dialectical behavior" OR "dialectical behavioural" OR "dialectical behavioral") TIAB Document title & abstract

Applied Limits: peer reviewed, English language

**PubMed Central (NCBI)**

((implement*[Title/Abstract]) OR (train*[Title/Abstract]) OR (workshop*[Title/Abstract]) OR (teach*[Title/Abstract]) OR (learn*[Title/Abstract]) OR (student*[Title/Abstract]) OR (develop*[Title/Abstract]) OR (educat*[Title/Abstract]) OR (course*[Title/Abstract]) OR (disseminat*[Title/Abstract]))

AND

(("dialectical behaviour" [Title/Abstract]) OR ("dialectical behavior"[Title/Abstract]) OR ("dialectical behavioural"[Title/Abstract]) OR ("dialectical behavioral"[Title/Abstract]))

Applied Limits: English language, pre-prints excluded.
