# Supplementary material for: The effectiveness of dialectical behaviour therapy training: a quantitative systematic review using Kirkpatrick’s four-level model
Source: Borderline Personal Disord Emot Dysregul. 2026 Apr 24;13:15. doi: 10.1186/s40479-026-00344-4 (PMC13244647; doi:10.1186/s40479-026-00344-4)
Supplement: Supplementary file 2 — Supplementary Material 2 [file 40479_2026_344_MOESM2_ESM.docx]

**Inclusion/Exclusion criteria**

| **PICO** | **Inclusion Criteria** | **Exclusion Criteria** |
| --- | --- | --- |
| **Population (P)** | - Therapists/clinicians/professionals, working in routine clinical services, who have participated in DBT training. - Service users whose outcomes are linked to therapist DBT training (where above criteria is met) | - Student or trainee only populations - Non-clinical services |
| **Intervention (I)** | - DBT training (workshops, courses, structured packages) intended to teach delivery/implementation of DBT. - Described training details (length, provider, content, format) - DBT extensions (DBT-PE, DBT-PTSD) | - Therapeutic DBT interventions or training with intentions other than to teach delivery/implementation of DBT - Supervision or CPD - Fundamentally different models (Radically Open DBT) |
| **Comparison (C)** | Level 1 (Reaction):   - No comparator (descriptive satisfaction) - Control group (trained vs. untrained)   Level 2 (Learning):   - Pre–post knowledge/skill/confidence/attitude - Format, duration, subgroup, benchmark, control, alternative training comparisons.   Level 3 (Behaviour):   - Pre–post behaviour/self-report - Format, fidelity benchmark, control, subgroup, alternative training comparisons.   Level 4 (Results):   - Pre–post-training service outcomes - Service-level, time-series, economic comparisons | - Comparisons unrelated to training (e.g., different therapies efficacy) - Studies without any training-related comparator or pre–post design when required for level |
| **Outcome (O)** | - Level 1: Trainee satisfaction, engagement - Level 2: Knowledge, attitudes, skills, confidence - Level 3: DBT skill use, adherence, burnout - Level 4: Client symptom change, implementation outcomes, organisational outcomes |  |
| **Study types** | - Quantitative, or mixed methods, with clear methods and extractable outcomes. Inferential statistics required for comparators. - Full-text, peer-reviewed, English-language articles | - Conference abstracts, case studies, discussion articles, dissertations, non–peer-reviewed and grey literature, protocols. |
