# Supplementary material for: The effectiveness of dialectical behaviour therapy training: a quantitative systematic review using Kirkpatrick’s four-level model
Source: Borderline Personal Disord Emot Dysregul. 2026 Apr 24;13:15. doi: 10.1186/s40479-026-00344-4 (PMC13244647; doi:10.1186/s40479-026-00344-4)
Supplement: Supplementary file 3 — Supplementary Material 3 [file 40479_2026_344_MOESM3_ESM.docx]

**Data extraction template**

| Reference |  | |
| --- | --- | --- |
| Country/Setting |  | |
| Study aim/purpose |  | |
| Methods | | |
| Type of study (Quantitative, mixed methods) |  | |
| Study Design  (e.g., pre–post, RCT, cross-sectional, retrospective cohort) |  | |
| Population- Staff | | |
| Number of participants |  | |
| Type (trainees/ professionals, service users if relevant), experience, context of work, demographics if reported |  | |
| Population- Clients | | |
| Number of participants |  | |
| Population |  | |
| Intervention | | |
| Training description-format (workshop, course), duration, provider (e.g., official DBT trainers), content focus | |  |
| DBT details (e.g. adapted, enhanced, skills-only) | |  |
| Training Context e.g. standalone training vs part of wider implementation, organisational support, prior DBT exposure if mentioned, funding, mandated/voluntary | |  |
| Comparators | |  |
| Outcomes according to Kirkpatrick’s model- outcome measures and timing of outcome | | |
| Level 1 |  | |
| Level 2 |  | |
| Level 3 |  | |
| Level 4 |  | |
| Relevant contextual factors (e.g. support systems, leadership, barriers, motivation, organisational readiness) |  | |
| Noted study limitations |  | |
| Conclusion/author recommendations |  | |
| Reviewers' comments |  | |
