# Supplementary material for: The effectiveness of dialectical behaviour therapy training: a quantitative systematic review using Kirkpatrick’s four-level model
Source: Borderline Personal Disord Emot Dysregul. 2026 Apr 24;13:15. doi: 10.1186/s40479-026-00344-4 (PMC13244647; doi:10.1186/s40479-026-00344-4)
Supplement: Supplementary file 4 — Supplementary Material 4 [file 40479_2026_344_MOESM4_ESM.docx]

**Examples of reasons for exclusion**

| **Citation** | **Relevance to review** | **Reasons for exclusion** |
| --- | --- | --- |
| Feigenbaum et al. (2012)  [86] | Implementation paper which involved providing mental health professionals with DBT training and assessing pre–post client outcomes. | No DBT training comparators: there were no comparators which would establish the specific effectiveness of the DBT training. Rather, the comparators in this study evaluate the effectiveness of DBT as an intervention in routine care. The study would have required pre- and post-training service outcomes to be considered for inclusion. |
| Hazelton et al. (2006)  [87] | The paper is an evaluation of DBT training for therapists in a mental health service and provides pre- and post-survey outcomes on staff knowledge, attitudes and experience of service users with BPD. | The paper only provides descriptive statistics in the form of percentages. No inferential statistics are calculated. |
| Clarke et al.  (2015)  [75] | The paper compared an ACT-based training intervention to a DBT skills-based training invention on staff attitudes. | The training was also a form of staff intervention, with the aim of reducing stigma and prejudice, not to train staff to deliver or implement DBT. |
| Carmel et al. (2019)  [48] | The paper compared DBT training to standard care training for psychiatric residents on attitudes, confidence and willingness to treat BPD. | The participants were all psychiatric residents, thus students, which met the exclusion criteria. |
| Navarro-Haro et a. (2019)  [78] | The paper prospectively evaluated predictors of adoption and reach of DBT in services following DBT-ITM. | There were no comparators for training effectiveness. The number of DBT modes implemented and clients reached were only evaluated at follow-up. The focus of the paper is the predictors of adoption and reach, which is related to implementation, rather than training effectiveness. |

**Supplementary File 4 References**

1. Feigenbaum JD, Fonagy P, Pilling S, Jones A, Wildgoose A, Bebbington PE. A real‐world study of the effectiveness of DBT in the UK National Health Service. Br J Clin Psychol. 2012;51(2):121-141. doi:10.1111/j.2044-8260.2011.02017.x
2. Hazelton M, Rossiter R, Milner J. Managing the ‘unmanageable’: Training staff in the use of dialectical behaviour therapy for borderline personality disorder. Contemp Nurse. 2006;21(1):120-130. doi:10.5172/conu.2006.21.1.120
3. Carmel A, Logvinenko E, Valenti ES. Evaluation of a dialectical behavior therapy psychiatry residency training program. Acad Psychiatry. 2019;43:37-40. doi:10.1007/s40596-018-0993-4
