# Supplementary material for: The effectiveness of dialectical behaviour therapy training: a quantitative systematic review using Kirkpatrick’s four-level model
Source: Borderline Personal Disord Emot Dysregul. 2026 Apr 24;13:15. doi: 10.1186/s40479-026-00344-4 (PMC13244647; doi:10.1186/s40479-026-00344-4)
Supplement: Supplementary file 5 — Supplementary Material 5 [file 40479_2026_344_MOESM5_ESM.docx]

**Quality Appraisal Results**

Below are the results of the quality appraisal. A range of tools were used as the review comprised studies of many designs, thus, appraisals are organised according to the quality appraisal tools used. Overall risk of bias was rated following GRADE guidance, using the researcher’s judgement, which considered how many elements of bias were at risk and how significant these were to the study results [32]. These were rated low, moderate (mod), or high. Some checklist items required a judgement for each outcome; where all outcomes had same answer this is presented as one judgement, otherwise it is noted where outcomes had mixed judgements.

**Quasi-Experimental Studies**

Quality Appraisals Using JBI Checklist for Quasi-Experimental Studies [29] and Overall Appraisal

|  | **TP** | **SA** | **CONF** | **AI** | **Assessment, Detection and Measurement of the Outcome** | | | **PR** | **SCV** | **Overall Risk of Bias** | **Strengths** | **Limitations** | **Notes** |
| --- | --- | --- | --- | --- | --- | --- | --- | --- | --- | --- | --- | --- | --- |
|  |  |  |  |  | **MM** | **MS** | **MR** |  |  |  |  |  |  |
| Ashworth et al. (2016)  [35] | Yes | No | Yes | Yes | Yes | Yes | No | Yes | Yes | High | Appropriate statistics for small sample | No control; self-report Likert scales; no reliability checks; no reporting of how measures conducted; poor reporting of descriptive data. |  |
| Bender et al. (2023)  [45] | Yes | No | No | Unclear | Yes | Yes | Mixed* | Yes | Yes | Mod | Clear pre–post design; multiple timepoints; good internal consistency for most measures; real-world context. | No control; self-report measures; knowledge measure had poor internal consistency; high attrition (approximately 50%) but analysed with no significant bias; low powered analysis (*n* =16). | *Knowledge- No  Other outcomes- Yes |
| Carmel et al. (2014)  [48] | Yes | No | No | Unclear | Yes | Yes | No | No | Yes | High | Validated burnout measure (CBI) used; real-world clinical setting. | No control; heterogeneous sample with possible service-level confounders not analysed; no measure reliability checks; large attrition with no analysis; small sample |  |
| Harned et al. (2021a)  [46] | Yes | No | Unclear | Unclear | Mixed* | Yes | Mixed** | Yes | Yes | Mod- High | Mostly validated, reliable measures; follow up measurements; large sample at baseline. | No control; no between group participant descriptions; post-measures only for Intended Use and Actual Use; self-report scales only; significant attrition, analysed between groups but not survey completers vs non-completers. | *Credibility- Yes  Self-efficacy- Yes  Concerns- Yes  Intended Use- no  Actual Use- No  Barriers- Yes  **Credibility- Yes  Self-efficacy- Yes  Concerns- Yes  Intended Use- Partial Yes  Actual Use- No  Barriers- No |
| Haynos et al. (2016)  [42] | Yes | No | Yes | Unclear | Yes | Yes | Mixed | Unclear | Yes | Mod | Mostly validated measures; explored correlations. | No control; no reliability measure for knowledge; heterogenous sample; concerns re knowledge test validity; incomplete follow-up reporting; correlation coefficients not reported; small sample. | * Knowledge- No  Burnout- Yes  Stigma- Yes |
| Herschell et al. (2014)  [38] | Yes | No | Yes | Unclear | Yes | Yes | Mixed* | Mixed** | Yes | Mod | Internal consistency assessed; attrition analysis for Use of DBT. | No control; measures not validated; Attitudes borderline reliability; high attrition (approximately 45%) and only partially analysed. | *Confidence- Yes  Use of DBT- Yes  Attitudes- Partial Yes  **Confidence- No  Use of DBT-  Partial Yes  Attitudes- No |
| Holbrook et al. (2022)  [37] | Yes | No | Yes | Unclear | Yes | Yes | Mixed* | Yes | Yes | Mod | Reliable turnover data from Human Resources. | No control; lack of confounders considered or described; knowledge measure not standardised or tests for reliability or conditions reported. | *Knowledge- No  Staff Turnover- Yes |
| McCay et al. (2017)  [34] | Yes | No | Yes | Unclear | Yes | Yes | No | No | Yes | Mod | Multiple outcome measurements at pre- and three post-training time points. | No control; self-report unvalidated measures; no tests for reliability; no reporting of knowledge test conditions; high attrition with no analysis; statistical test appropriate but limited. |  |
| Navarro-Haro et al. (2024)  [43] | Yes | No | Yes | Unclear | Yes | Yes | Yes | Mixed* | Yes | Mod | Reliable outcome measures with good internal consistency; consideration of baseline differences and some confounders. | No control; heterogenous sample; some confounders assessed but not all; significant attrition, while analysed for most outcomes, significant differences found; no attrition analysis for implementation. | *Confidence- Yes  Self-efficacy- Yes  Concerns- Yes  Burnout- Yes  Implementation- No |
| Pasieczny & Connor (2011)  [55] | Yes | Yes | Yes | No | Yes | Mixed* | Yes | Yes | Yes | Low-Mod | Intention-to-treat (ITT) analysis used included missing data and no bias found; high follow-up rates (93% DBT, 87% TAU); self-report measures had excellent internal consistency; hospital records for clinical outcomes verified through interviews. | Unequal treatment exposure; no controlling of medication; self-report data for only half of the sample. | *Self report measures- No  All other measures- Yes |
| Perseius et al. (2007)  [47] | Yes | No | Yes | Unclear | Yes | Yes | Yes | No | Yes | Mod | Standardised scale with good internal consistency. | No control; small sample; high attrition with no analysis; self-report scales. |  |
| Tan et al. (2023)  [44] | Yes | Yes | Yes | No | Yes | Yes | No | Yes | Yes | Mod | Matched control; standardised measures. | Not analysed but noted differences in clients and environment between groups; small sample; self-report measures with no checks for internal consistency. |  |
| Tebbett-Mock et al. (2020)  [53] | Yes | No | Unclear | Unclear | Yes | Yes | Mixed* | Yes | Unclear | Mod | Clear intervention and comparator, same setting with similar groups, valid outcome measurement, appropriate stats for client outcomes. | No follow-up, no control group, retrospective hospital data, no repeated measures, small effect sizes, simplistic analysis of cost savings, excluded clients with a learning disability diagnosis (uneven across groups), not randomised, no tests for normality reported (methodological rigor issue). | *Client outcome- Unclear  Cost- Yes |
| Tebbett-Mock et al. (2021)  [54] | Yes | No | Unclear | Unclear | Yes | Yes | Unclear | Yes | Unclear | Mod | Same setting and similar participants, valid outcome measurement. | Retrospective hospital data, no control group, no repeated measures, possible bias due to exclusions, small effect sizes, no report of tests for normality or whether adjusted for multiple comparisons. |  |

*Note.* Many included studies were conducted in routine setting and used pre–post designs without control or comparison groups. As such, the quality appraisal item related to similarity of care outside the intervention, was commonly rated as 'Unclear.' Unless explicitly stated, it was not possible to determine whether other changes or interventions occurred during the study period that may have influenced outcomes.

TP = Temporal Precedence; SA = Selection/Allocation; CONF = Confounders; AI = Administration of Intervention/Exposure.; MM = Multiple Measures; MS = Measured Same Way; MR = Measured in a Reliable Way; PR = Participant Retention; SCV = Statistical Conclusion Validity

**Cross-Sectional Studies**

Quality Appraisals Using JBI Checklist for Analytical Cross-Sectional Studies [28] and Overall Appraisal

|  | | **Clearly defined inclusion criteria** | | **Participants and setting clearly defined** | | **Exposure measurement reliability and validity** | | **Objective, standard criteria for measurement of the condition** | | **Identified confounding factors** | | **Strategies to deal with confounders** | | **Outcome measurement reliability and validity** | | **Appropriate statistical analysis** | | **Overall risk of bias** | | **Strengths** | | **Limitations** | |
| --- | --- | --- | --- | --- | --- | --- | --- | --- | --- | --- | --- | --- | --- | --- | --- | --- | --- | --- | --- | --- | --- | --- | --- |
| Harned et al. (2021b)  [49] | | Yes | | Yes | | Yes | | Yes | | No | | No | | Yes | | Unclear | | Moderate | | Detailed demographics; validated; reliable adherence measure (DBT ACS). | | Therapist DBT training not clearly described or confirmed; no identification or control of potential confounders; no clear statistical test specified for benchmark comparison. | |
| Hawkins & Sinha (1998) | | Yes | | No | | No | | Yes | | Yes | | Yes | | No | | Yes | | Moderate | | Large sample; real world context; DBT specific exam and validation efforts; multiple predictors assessed. | | Limited demographic data; self-reported training, not a standarised measure of DBT knowledge; no test re-test reliability; inconsistent testing conditions; pre post analysis is not large sample/powered robustly. | |
| Harned et al. (2024)  [50] | | Yes | | Yes | | No | | No | | No | | Yes | | No | | No | | Moderate | | Validated outcome measure; reliable coding. | | No training detail; no confounder control; reported client differences; incorrect statistical method (t-tests did not account for clustered data). | |
| DiGiorgio et al. (2010)  [51] | | Yes | | Yes | | Yes | | No | | No | | No | | No | | Yes | | High | | Demographic details; double coding with good inter-rater reliability; some exposure validation via recruitment source. | | Training not objectively measured; reliance on self-report for key variables; poor inaccurate measure of adherence; comparator groups not clearly defined or comparable; missing information on client demographics per group. | |

**Randomised Controlled Trials**

Quality Appraisals Using JBI Checklist for Randomised Controlled Trials [27]

|  | **Bias related to selection and allocation** | | | **Bias related to administration of intervention/exposure** | | | **Bias related to assessment, detection and measurement of the outcome** | | | **PP**  **retention** | **Statistical Conclusion Validity** | | | **Notes** |
| --- | --- | --- | --- | --- | --- | --- | --- | --- | --- | --- | --- | --- | --- | --- |
|  | **Randomised** | **Concealed** | **Similar** | **PP’s blind** | **Delivery blind** | **Identical treatment** | **Assessors blind** | **Measured same** | **Reliable measure** |  | **Analysed in groups** | **Appropriate analysis** | **Appropriate design** |  |
| Dimeff et al. (2009)  [39] | No | No | Yes | No | No | No | Unclear | Mixed* | Mixed** | No | No | Yes | Yes | * Skills use- Yes  All other outcomes-No  **Training Evaluation, Confidence/Motivation, Role plays- Yes  All other measures- No |
| Dimeff et al. (2011)  [40] | No | Unclear | Yes | No | No | Yes | Unclear | Yes | Mixed* | No | Yes | Yes | Yes | *Knowledge and Skills Use- No  All other outcomes- Yes |
| Dimeff et al. (2015)  [41] | No | Unclear | Yes | Yes | No | Yes | Unclear | Mixed* | Mixed** | No | Partial Yes | Yes | Yes | *Skills use- Yes  All other outcomes- No  **Clinical Use and Barriers- No  All other outcomes- Yes |

*Note.* PP = participant.

RCT Overall Appraisal

|  | **Overall Risk of Bias** | **Strengths** | **Limitations** |
| --- | --- | --- | --- |
| Dimeff et al. (2009)  [39] | High | Similar baseline characteristics; design appropriate; role play outcome measures had strong inter-rater reliability with coding; attrition rates were similar across groups. | Not true randomisation, allocation minimisation not purely random allocation concealment was not described or not concealed; participants and interventionists were not blinded; timing of outcome measurement differed between groups; self-report measures- only some had reliability checks; attrition impacts were not fully analysed; analysis did not adhere to intention-to-treat; randomised participants were excluded post-randomisation but before baseline. |
| Dimeff et al. (2011)  [40] | Moderate | Similar baseline characteristics; similar treatment time and exam conditions; most outcomes had high internal consistency; assessments conducted blind. | Not true randomisation, allocation minimisation not purely random allocation concealment was not described; not clear whether scoring was blind; Knowledge and Skills-Use lacked reliability measures; incomplete follow-up and no analysis of attrition. |
| Dimeff et al. (2015)  [41] | Moderate | Similar baseline characteristics; participants were blinded at first assessment; most measures were assessed for reliability; training time and conditions was consistent across groups. | Not true randomisation, allocation minimisation not purely random allocation concealment was not described; not clear whether scoring was blind; timing of outcome assessment differed between groups; some key outcome lacked reliability checks; high and uneven attrition; modified ITT excluded some randomised participants. |

**Cohort Studies**

Quality Appraisals Using JBI Checklist for Cohort Studies [28]

|  | **Similar groups** | **Exposure measured similarly** | **Exposure measurement reliable and valid** | **Confounders identified** | **Strategies to deal with confounders** | **Pp’s free of outcome at the start** | **Outcomes measures reliable and valid** | **Follow-up time** | **Follow-up complete or explored** | **Strategies for incomplete follow-up** | **Appropriate statistical analysis** |
| --- | --- | --- | --- | --- | --- | --- | --- | --- | --- | --- | --- |
| Trupin et al. (2002)  [52] | No | Yes | No | Yes | Yes | No | No | Yes | No | No | Yes |
| King et al. (2018)  [56] | Unclear | Yes | Yes | No | No | Yes | Yes | Yes | No | Yes | Yes |

Cohort Studies Overall Appraisal

|  | **Overall Risk of Bias** | **Strengths** | **Limitations** |
| --- | --- | --- | --- |
| Trupin et al. (2002)  [52] | High | Various levels of training allowed for some comparison of training effects; standardised risk assessment tools | Retrospective design; significant baseline differences; DBT training not well described and no measure of adherence; some outcomes (e.g. staff punitive behaviours) relied on behaviour logs that authors reported could be used inconsistently; incomplete statistical comparisons between groups. |
| King et al. (2018)  [56] | Moderate | Large sample over long time period; standardised database; strategies accounted for missing data. | Differences in censored data between cohorts; small sample for inactive teams; no control or exploration of confounding variables (e.g. staffing, location, setting); lack of exploration of missing data. |

**Descriptive Studies**

Quality Appraisals Using CASP Checklist Descriptive/Cross Sectional Studies [31]

|  | **Clear focus** | **Method answered question** | **Recruitment acceptable** | **Accurate measures** | **Data addressed research issue** | **Enough participant for analysis** | **Results presentation** | **Data analysis rigor** | **Clear statement of findings** | **Generalisability** | **Research value** |
| --- | --- | --- | --- | --- | --- | --- | --- | --- | --- | --- | --- |
| Kauth et al. (2017)  [30] | Yes | No | No | No | No | Yes | No | No | No | No | No |

Descriptive Studies Overall Appraisal

|  | **Overall Risk of Bias** | **Strengths** | **Limitations** |
| --- | --- | --- | --- |
| Kauth et al. (2017)  [30] | High | Descriptive training satisfaction and user experience; insight into perceived usefulness of facilitation components. | No pre–post or comparator, purely descriptive; self-selected, likely highly motivated sample; self-report data only; no objective outcome measures which did not address research aim; survey details (items, validation) not reported; analysis limited to descriptive statistics (percentages). |
