# Supplementary material for: The effectiveness of dialectical behaviour therapy training: a quantitative systematic review using Kirkpatrick’s four-level model
Source: Borderline Personal Disord Emot Dysregul. 2026 Apr 24;13:15. doi: 10.1186/s40479-026-00344-4 (PMC13244647; doi:10.1186/s40479-026-00344-4)
Supplement: Supplementary file 6 — Supplementary Material 6 [file 40479_2026_344_MOESM6_ESM.docx]

**GRADE Assessments**

In line with the JBI methodology for effectiveness reviews [25], certainty of evidence was assessed for six key outcomes: knowledge, confidence/self-efficacy, adherence, use of DBT in clinical practice, implementation outcomes, and suicide/self-harm. The GRADE approach [32] was used to evaluate the overall confidence in the effects for each outcome. GRADE supports decision-making by summarising the strength and reliability of evidence across studies. Evidence is initially rated based on study design (e.g., RCTs are rated as high certainty) and may be downgraded or upgraded based on five domains: risk of bias, inconsistency (unexplained heterogeneity), indirectness (relevance), imprecision (e.g., wide confidence intervals or small sample sizes), and publication bias.

**Certainty of Evidence for Pre–Post Changes Related to DBT Training**

| **Outcome** | **No. of Participants** | **No. of Studies** | **Follow-up duration** | **Outcome measure/instrument** | **Effect Estimate** | **Certainty of Evidence** | **Comments/Explanation** |
| --- | --- | --- | --- | --- | --- | --- | --- |
| DBT knowledge acquisition | 125 | 5 | Up to 12-16 months post-training | Various direct knowledge assessments (e.g., DBT Skills Knowledge Test or adapted versions), and one Likert-style self-report. | All studies reported post-training improvements in knowledge. One study reported large effect sizes (*d*=1.53–1.56). | ⨁◯◯◯  Very low (due to risk of bias, imprecision) | Downgraded x2. Moderate-high risk of bias mainly from lack of control groups, lack of reliability checks for knowledge measures, attrition issues; some imprecision due to small samples and wide variation, most did not report effect sizes or confidence intervals. |
| Confidence and self-efficacy in using DBT | 575 | 5 | Up to 12-16 months post-training | Behavioural Anticipation and Confidence Questionnaire (BAQ; 2), plus study-designed scales (3). | Consistent post-training improvements; one study large effects reported (*d* = 1.36–1.68). | ⨁◯◯◯  Very low (due to risk of bias, imprecision) | Downgraded x2. Moderate-high risk of bias from no control groups, self-report measures, some lacking reliability assessment; imprecision due to small samples and wide variation, most did not report effect sizes or confidence intervals. |
| Use of DBT in clinical practice | 91 | 3 | Up to 8 months post-training | Self-report use of DBT, one reported perceived behaviour changes on Likert scale. | Inconsistent post-training outcomes; two studies with intensive trainings reported training increased DBT behaviours; effect sizes ranged from small to moderate (*d* = 0.30–0.61). One day LD adaption training did not show change. | ⨁◯◯◯  Very low (due to risk of bias, imprecision) | Downgraded x 2. Moderate-high risk of bias due to no control group, self-report measures, poor or no reliability checks. Imprecision risks due to small sample with high attrition not analysed. |
| Implementation outcomes | 61 | 1 | Post DBT-ITM only. | Program Elements of Treatment Questionnaire | Significant pre- to post-training increases in group skills training, consultation team use, phone coaching, and mindfulness, with mindfulness showing the most consistent improvement. No significant change in the use of individual DBT therapy. | ⨁◯◯◯  Very low (due to risk of bias, imprecision) | Downgraded x2. Single observational study with high attrition, baseline subgroup differences, and potential confounding. Despite statistically significant findings, small follow-up sample limits confidence in precision and generalisability. |
| Client suicide and self-injury outcomes. | 1,194 (clients) | 2 | Up to 6 months post-treatment. | Retrospective hospital data | Mixed outcomes, possibly depending on training. Reduced self-harm and suicide following well-trained team, though small effect (*r* = .07 –.10). Basic-trained team had no effect on suicide attempts and increased self-injury (*E²* =.05). | ⨁◯◯◯  Very low (due to risk of bias, imprecision, indirectness) | Downgraded x3. Moderate risk of bias due to retrospective data with no repeated measures, lack of control group and non-randomised designs. Directness and imprecision concern due to uncontrolled confounding variables and small effect sizes. |
| *Note.* No pre–post studies measured adherence. | | | | | | | |

**Certainty of Evidence for the Effectiveness of DBT Training compared to No DBT Training**

| **Outcome** | **Comparator** | **No. of Participants** | **No. of Studies** | **Follow-up duration** | **Study design** | **Outcome measure** | **Effect Estimate** | **Certainty of Evidence** | **Comments/Explanation** |
| --- | --- | --- | --- | --- | --- | --- | --- | --- | --- |
| DBT knowledge acquisition | No training | 109 | 1 | - | Cross-sectional | Study designed exam. | Trained clinicians scored higher than untrained. | ⨁◯◯◯  Very low (due to risk of bias, imprecision) | Downgraded x2. Moderate risk of bias from limited demographic data, non-standardised DBT exam, inconsistent testing conditions, no reliability measure. Imprecision concerns due to one study, with no effect sizes, and analysis not robustly powered. |
|  | Self-directed manual | 454 | 3 | Three months | RCTs | DBT skills knowledge and application test | DBT skills training (especially e-learning) showed greater knowledge gains than manual, with medium effect sizes (d = 0.36–.62). | ⨁⨁◯◯  Low (due to risk of bias) | Downgraded x2. Moderate-high risk of bias due to not true randomisation, concealment not described; not clear whether scoring was blind; Knowledge lacked reliability measures; incomplete follow-up and no analysis of attrition. |
|  | e-Control | 89 | 1 | 15 weeks | RCTs | DBT skills knowledge and application test | e-DBT outperformed e-control with very large effect sizes (*d* =3.48, 1.90). | ⨁⨁⨁◯  Moderate (due to risk of bias) | Downgraded x1. Moderate risk of bias due to not true randomisation, concealment not described; not clear whether scoring was blind; Knowledge lacked reliability measures; incomplete follow-up and no analysis of attrition. |
| Confidence and self-efficacy in using DBT | Self-directed manual | 454 | 3 | Three months | RCTs | Behavioural Anticipation and Confidence Questionnaire (2); study designed measure (1) | Some evidence of a short-term improvement in confidence for e-learning and instructor-led training compared to manual (1 study, *d* = 0.54–0.68), but no evidence of sustained effects and effects were not consistent across studies. | ⨁⨁◯◯  Low (due to risk of bias, inconsistency) | Downgraded x2. Moderate risk of bias due to not true randomisation, concealment not described; not clear whether scoring was blind; incomplete follow-up and no analysis of attrition. Inconsistency concerns as short-term improvement not seen across studies. |
|  | e-Control | 89 | 1 | 15 weeks | RCT | Study designed measure | e-DBT outperformed e-Control (*d* =0.76, 0.83). | ⨁⨁⨁◯  Moderate (due to risk of bias) | Downgraded x1. Moderate risk of bias due to not true randomisation, concealment not described; not clear whether scoring was blind; incomplete follow-up and no analysis of attrition. |
| Use of DBT in clinical practice | Self-directed manual | 454 | 3 | Three  months | RCTs | Self-report use of DBT skills. | Inconsistent results; two studies found no differences, one study found e-DBT led to higher skills use than manual. | ⨁◯◯◯  Very low (due to risk of bias, inconsistency) | Downgraded x 3. Moderate-high risk of bias due to not true randomisation, concealment not described; not clear whether scoring was blind; self-report and lacked reliability measures; incomplete follow-up and no analysis of attrition. Inconsistent effect. |
|  | e-Control | 89 | 1 | 15  weeks | RCT | Self-report use of DBT skills. | e-DBT led to consistently higher skills use than e-Control. | ⨁⨁⨁◯  Moderate (due to risk of bias) | Downgraded x1. Moderate risk of bias due to not true randomisation, concealment not described; not clear whether scoring was blind; incomplete follow-up and no analysis of attrition. |
| Adherence | Self-directed manual | 322 | 2 | Three  months | RCTs | Performance-based role plays coded. | No significant difference between DBT skills training (e-learning or instructor led) and manual learning. | ⨁⨁⨁◯  Moderate (due to risk of bias) | Downgraded x1. Moderate risk of bias due to not true randomisation, concealment not described; not clear whether scoring was blind; incomplete follow-up and no analysis of attrition. Indirectness caution as role-plays may not reflect adherence in clinical settings. |
| *Note.* No studies comparing DBT training to a control reported outcomes on implementation, or client suicide and self-injury. While bias was not evident enough to contribute to a downgrade of evidence, it should be noted that all RCTs by Dimeff et al. may be at risk of publication bias due to the authors’ affiliation with a DBT training company.  DBT-ITM = The DBT Intensive Training™ Model  RCT= Randomised Controlled Trial | | | | | | | | | |

**GRADE Assessment: Certainty of Evidence for the Effectiveness of Intensive DBT Training compared to Non-Intensive Training**

| **Outcome** | **No. of Participants** | **No. of Studies** | **Study design** | **Outcome measure/instrument** | **Effect Estimate** | **Certainty of Evidence** | **Comments/Explanation** |
| --- | --- | --- | --- | --- | --- | --- | --- |
| DBT knowledge acquisition | 109 | 1 | Cross-sectional | Study designed exam | Five-day workshop completers scored higher than non-attendees, who also had more previous training. Reading DBT materials (*r* = .59), peer consultation (*r* = .63), and study group attendance (*r* = .65) strongly predicted knowledge; combined training types led to higher scores. | ⨁◯◯◯  Very low (due to risk of bias, imprecision) | Downgraded x2. Moderate risk of bias from limited demographic data, non-standardised DBT exam, inconsistent testing conditions, no reliability measure. Imprecision concerns due to one study, with no effect sizes, and analysis not robustly powered. |
| Use of DBT in clinical practice | 129 | 1 | Cross-sectional | Self-report use of DBT components | DBT-ITM therapists more frequently used skills groups (*d* =0.41), consultation teams (*d* = 0.34)*, phone consultations (*d* = 0.30)*, diary cards/homework (77.5% vs. 55.4%), and target hierarchy (55% vs. 21.4%) than non-DBT-ITM therapists. | ⨁◯◯◯  Very low (due to risk of bias) | Downgraded x 2. One study with high risk of bias due to training not objectively measured; reliance on self-report; comparator groups not clearly defined or comparable; missing information on client demographics per group. |
| Adherence | 129 | 1 | Cross-sectional | Self-reported DBT component adaptions subtracted from self-reported use of components | No significant difference between therapists who had attended DBT-ITM vs other trainings. | ⨁◯◯◯  Very low (due to risk of bias, indirectness) | Downgraded x3. Single study with high risk of bias due to training not objectively measured; comparator groups not clearly defined or comparable; missing information on client demographics per group. Imprecision concern due to invalid measure of adherence. |
| Client suicide and self-injury outcomes. | 1,194 (clients) | 2 |  | Retrospective hospital data | Mixed outcomes, possibly depending on training. Reduced self-harm and suicide following well-trained team, though small effect (*r* = .07 –.10). Basic-trained team had no effect on suicide attempts and increased self-injury (*E²* = .05). | ⨁⨁◯◯  Low (due to observational studies) | No downgrade. Risk of bias low-moderate, lowest in highest effect estimate. Consistent pattern of effects and decent sample sizes. |
| *Note.* No studies comparing intensive DBT training to non-intensive training reported outcomes on confidence and self-efficacy, or implementation.  DBT-ITM = The DBT Intensive Training™ Model  RCT= Randomised Controlled Trial | | | | | | | |
